# Reported Distribution, Species Richness, and Sampling Bias of *Culicoides* (Diptera: Ceratopogonidae) in Türkiye: A Literature-Based Synthesis with a Biogeographical Framework

**DOI:** 10.3390/insects17080858

**Published:** 2026-08-17

**Authors:** Gamze Pekbey, Güngör Karakaş

**Affiliations:** 1 Department of Plant Protection, Faculty of Agriculture, Yozgat Bozok University, 66100 Yozgat, Türkiye; 2 Department of Agricultural Economics, Faculty of Agriculture, Yozgat Bozok University, 66100 Yozgat, Türkiye; gungor.karakas@bozok.edu.tr

**Keywords:** biting midges, Wallacean shortfall, survey coverage, beta diversity, vector surveillance, PRISMA, SDG 15 (Life on Land)

## Abstract

*Culicoides* biting midges are important veterinary vectors, yet reported distributional patterns in Türkiye may reflect both ecological structure and uneven sampling. We synthesized 58 eligible Türkiye-specific reports (50 database-derived and eight citation-traced), consolidated them into 43 study/data-source families, and compiled 1164 taxon records representing 62 taxa. The all-explicit province dataset included 33 provinces and 45 taxa, whereas the primary province dataset included 18 provinces and 43 taxa. National taxonomic sources supported 72 species within the eligible evidence chain and 74 under a later taxonomic interpretation. Reported richness was strongly associated with sampling effort (Poisson coefficient = 0.579, 95% CI: 0.385–0.773), whereas biogeographical region was not significant after effort was accounted for (likelihood-ratio *p* = 0.914). Jaccard PERMANOVA identified an exploratory regional compositional pattern (F = 4.388, R^2^ = 0.369, *p* = 0.001; PERMDISP *p* = 0.325), with turnover accounting for approximately 78% of mean between-region dissimilarity. Most richness diagnostics ranged from 54 to 64 taxa, whereas Chao2 was unstable (69.6; 95% CI: 49.5–152.0). Vector-related evidence remained insufficient for species–pathogen risk modeling. Overall, the synthesis is consistent with a Wallacean shortfall strongly associated with uneven sampling and supports standardized, biogeographically balanced surveillance to test transition-zone structure, climate-sensitive phenology and redistribution, and integrated vector-host-pathogen hypotheses under comparable sampling effort.

## Simple Summary

Biting midges of the genus *Culicoides* are important in veterinary entomology, but published evidence on their distribution in Türkiye is uneven across provinces and biogeographical regions. This review organizes the literature into linked evidence layers covering eligible reports, national taxonomy, locality- and province-level occurrences, assemblage composition, and vector-related findings. Reported *Culicoides* richness is strongly shaped by unequal sampling: well-studied provinces have more recorded species, whereas poorly sampled areas may appear species-poor because they remain under-documented. Regional assemblages show broad compositional differences, but the literature-derived data cannot determine whether climate, elevation, livestock distribution, habitat, or other environmental factors drive these patterns. Vector-related evidence is likewise too sparse and heterogeneous for reliable species–pathogen risk rankings or predictive maps. The available evidence instead identifies where surveillance is strongest and where major gaps remain. Future work should use standardized, seasonally replicated sampling across Türkiye’s biogeographical sectors and transition zones, combine morphological and molecular identification, and integrate vector abundance with host-feeding, pathogen, weather, habitat, and livestock data. Coordinated long-term surveillance would help distinguish true ecological patterns from uneven documentation and test climate-sensitive changes in *Culicoides* distribution, phenology, and vector-related risk.

## 1. Introduction

Biting midges of the genus *Culicoides* Latreille, 1809 (Diptera: Ceratopogonidae), are small hematophagous flies of major veterinary importance. Their importance stems primarily from their roles as demonstrated or putative vectors of viruses affecting domestic and wild animals, including bluetongue virus (BTV), epizootic hemorrhagic disease virus, African horse sickness virus, Akabane virus (AKAV), Schmallenberg virus (SBV), and other arboviruses [1,2]. Although more than 50 viruses have been isolated from *Culicoides* species worldwide, true vector competence has been experimentally demonstrated for only a limited number of taxa [1,2]. Field detections therefore require cautious interpretation, particularly when viral RNA is detected in whole-body pools rather than in dissected salivary tissues or experimentally infected individuals [2]. Effective arbovirus risk assessment and outbreak preparedness depend on knowing which species are present, where they occur, and in what abundance; distribution and richness data contribute directly to risk maps, vector-free period definitions, and surveillance targeting [3]. Where species inventories are incomplete, or sampling effort is uneven, both vector-status assignments and transmission-risk estimates can be seriously biased [4,5].

Türkiye lies at the intersection of Europe, Asia, and the Middle East and spans the Euro-Siberian, Mediterranean, and Irano-Turanian biogeographical regions. Its Mediterranean, continental, semi-arid, coastal, and mountainous environments provide diverse habitats for *Culicoides* species. Wind-assisted dispersal is epidemiologically relevant across the broader region [6], and field studies from Türkiye document *Culicoides*–arbovirus associations [7]. Historical analyses suggest that wind-assisted movement of infected midges may have contributed to the introduction of bluetongue and Akabane viruses into western Türkiye [8]; similar modeling approaches have been proposed to quantify *Culicoides* dispersal and bluetongue spread across the Mediterranean and Balkan regions [6].

The first comprehensive review of the Turkish *Culicoides* fauna documented 57 species from 54 localities, establishing a baseline for national species diversity and distribution [9]. An updated checklist, based on entomological surveillance in 51 provinces and 104 sampling stations together with a critical review of previous records, increased the reported national total to 71 species and added ten new country records [10]. A subsequent record of *C. clastrieri* from Sinop Province raised the reported fauna to 72 species [11]. A national faunistic monograph [12] later recognized two additional species through retrospective taxonomic reassessment, bringing the contextual total to 74, as discussed in Section 4.1. Regional studies have since improved representation of previously under-sampled areas, particularly the Black Sea Region [13,14], while ecological studies have added information on local assemblages [15].

Despite the growing literature, the distribution of records remains uneven [9,10,13,14]. Some provinces and regions have been repeatedly sampled, while others are poorly represented or lack structured province-level data. In addition, vector-related studies vary in design, taxonomic resolution, and interpretation [15,16,17]. Pathogen detection, host-feeding evidence, and outbreak-associated vector surveys provide valuable information, but they do not all support the same level of inference regarding vector competence [2,16,17]. This unevenness has direct surveillance implications: incomplete species inventories and spatially biased records limit the ability to construct reliable risk maps, identify high-risk areas and seasons, and evaluate vector assemblage composition across ecological zones [3,18]. Such coverage-driven gaps exemplify the Wallacean shortfall, in which apparent absences may reflect under-sampling rather than true distributional limits [19].

This review systematically compiles published *Culicoides* records from Türkiye using a PRISMA-guided quantitative framework. The objectives were as follows: (i) summarize the historical development of reported species richness and the temporal accumulation of the eligible evidence corpus; (ii) define the hierarchy linking the literature corpus, national taxonomic totals, taxon records extracted at locality, province, or habitat resolution, province-level incidence matrices, and vector evidence; (iii) evaluate province- and region-level reported richness and sampling gaps; and (iv) assess whether reported richness and evidence coverage differ among the three biogeographical regions after accounting for sampling effort. The synthesis further aimed to achieve the following: (v) explore assemblage composition while explicitly delimiting the scope of mechanism-level inference; (vi) synthesize vector-related findings through an evidence-gap framework; and (vii) translate sampling, transition-zone, biogeographical, and vector-evidence patterns into explicit, testable hypotheses for standardized future surveillance.

## 2. Materials and Methods

### 2.1. Study Scope

This study was designed as a literature-based quantitative synthesis of *Culicoides* records from Türkiye, with emphasis on reported species richness, sampling bias, and surveillance gaps relevant to arbovirus monitoring. The complete eligible evidence corpus covered publications from 1918 to 2026. Because the literature search was completed in August 2026, the publication-trend analysis was restricted to completed calendar years through 2025; the incomplete 2026 calendar year was not compared directly with earlier full years. Eastern Mediterranean epidemiological context was considered when interpreting field pathogen detection and vector-related evidence, particularly for BTV, AKAV, SBV, and bovine ephemeral fever virus (BEFV) [7,15,16,20,21].

The taxonomic scope was limited to *Culicoides* species and species complexes reported in faunistic, ecological, molecular, host-feeding, and arbovirus-related studies. Records from other Ceratopogonidae genera were excluded from the main species richness dataset, even when they were included in broader family-level studies, to maintain a clear focus on *Culicoides*.

### 2.2. Literature Selection and Data Extraction

Published studies were screened for *Culicoides* species occurrence and other original Türkiye-specific *Culicoides* evidence. The literature search was conducted in Web of Science, Scopus, and Google Scholar using the expression “*Culicoides*” AND (“Turkey” OR “Turkiye”), with no date restriction; searches were completed in August 2026. A total of 370 records/candidates were identified across the three sources (Web of Science, n = 94; Scopus, n = 57; Google Scholar, n = 219). After removal of 139 duplicate or already represented records, 231 records underwent title-and-abstract screening, and 175 were excluded. Fifty-six reports were retrieved and assessed at full-text/report level; six were excluded, leaving 50 database-derived eligible reports. Full-text/report exclusions comprised secondary review, synthesis, or background reports (n = 5) and one report without demonstrated new Türkiye-specific *Culicoides* evidence (n = 1). Reference and citation searching identified eight additional original reports that met the same eligibility criteria, yielding the complete eligible evidence corpus of 58 reports. Initial title-and-abstract screening and full-text/report eligibility assessment were performed by the first author against predefined inclusion and exclusion criteria; the second author subsequently and independently verified the study-selection decisions at both stages, and disagreements were resolved by discussion until consensus. Publication type was not itself an exclusion criterion; journal articles, theses, book chapters, conference contributions, and other scholarly reports were assessed under the same evidence-based rule. Reports were eligible when they provided extractable original Türkiye-specific *Culicoides* evidence relevant to occurrence or distribution, ecology or breeding habitat, taxonomy or molecular characterization of Turkish material, pathogen screening or detection in field-collected *Culicoides*, host-feeding evidence, or original analysis of Türkiye-specific *Culicoides* data. Multiple eligible reports describing the same underlying field program, specimen series, or dataset were retained at report level for provenance but consolidated into 43 operational study/data-source families to prevent double counting (Supplementary File S4). One later national faunistic monograph [12] was used only for national taxonomic reconciliation and was not included in the eligible evidence-report corpus or province-level incidence matrices. No AI-assisted or other automation tool was used for searching, screening, eligibility decisions, or source-data extraction. The search strategy and harmonized screening counts are documented in Supplementary File S1 (Sheet S12), and the complete study-selection process is shown in the PRISMA 2020 flow diagram (Supplementary File S2) [22].

To avoid comparing an incomplete calendar year with completed years, the temporal analysis used the 57 eligible reports published between 1918 and 2025. Reports were grouped by publication year into calendar-aligned 5-year intervals, with a final 2020–2025 interval, and assigned to one of three broad descriptive categories according to their principal evidence focus: faunistic/ecological/taxonomic, vector/pathogen/host-feeding, or molecular/analytical. Because the terminal interval spans six rather than five years, report counts were divided by interval duration and expressed as eligible reports per year; cumulative totals were calculated from raw report counts. One eligible report was published in 2026 and is included in the complete 58-report evidence corpus but excluded from the completed-year temporal analysis because 2026 was incomplete when the search was completed in August 2026. These temporal summaries are descriptive and were not interpreted as standardized research effort or as ecological or epidemiological trends.

Eligible reports were designated as principal analytical sources when they provided original species-level occurrence records with extractable province- or locality-level information suitable for richness and mapping analyses. For national taxonomic reconciliation, the historical review of Turkish *Culicoides* [9] served as a synthesis benchmark; historical occurrence evidence recovered through that review was reassigned, where possible, to the cited original report(s) in the provenance layer, and the review itself was not treated as an analytical evidence-study identity. The updated checklist [10] and the subsequent new country record of *C. clastrieri* [11] establish a working total of 72 reported species in Türkiye within the eligible evidence chain. The later national faunistic monograph [12] recognizes *C. indistinctus* and *C. vitreipennis* through retrospective taxonomic reassessment, producing an updated contextual total of 74. These national taxonomic totals are not interchangeable with the 62 taxa represented in the taxon-record compilation, the 43 taxa in the primary province incidence matrix, or the 45 taxa in the all-explicit province dataset. Turgut [11] also provided the single province-level occurrence record of *C. clastrieri* from Sinop but was not counted among the ten principal analytical source-level survey units summarized in the study-design audit. Accordingly, this single-record taxonomic update was retained in the taxon-record and national-reconciliation layers but was not added to the all-explicit province dataset.

The extracted analytical data are provided in Supplementary File S1, the 58 eligible reports and their consolidation into 43 operational study/data-source families are documented in Supplementary File S4, and the separate taxonomic monograph [12] is identified in the main text and reference list. Supplementary File S4 also provides report-level provenance, eligibility decisions, and study-family assignments for the evidence corpus.

For each eligible record, the following information was extracted when available: reference, year, province, geographical region, locality or sampling station, species or species complex, specimen number, sampling method, study type, pathogen or host-feeding evidence, and notes on new records or taxonomic uncertainty. Extraction fields were retained even when a value was unavailable so that missing trap-night, duration, abundance, spatial-resolution, and identification information remained visible rather than being imputed. The complete extracted data are provided in Supplementary File S1 (Sheet S5).

Separately from the PRISMA-guided Türkiye-specific screening described above, targeted searches of Web of Science and Scopus, supplemented by citation tracing of retrieved inventory sources, were conducted to identify published *Culicoides* inventories from countries neighboring Türkiye and other Western Palaearctic and Mediterranean faunas for contextual discussion only (Section 4.4). Search combinations used “*Culicoides*”, “Ceratopogonidae”, and “biting midge” together with the names of countries and adjoining regions. Retrieved sources were classified as follows: (i) national or broad regional inventories supporting a defensible species total; or (ii) localized, single-survey, host-focused, or disease-focused studies documenting only part of a fauna, even when they discussed cumulative country counts. Both categories informed the assessment of evidence scope, but only category (i) was used for the comparative cross-border totals presented later in the Discussion. Combining scattered regional surveys with national totals would have created an asymmetric comparison and was therefore avoided. These contextual searches were not assessed against the Türkiye-specific eligibility criteria, were not represented in the PRISMA flow diagram or Supplementary File S4, and were not incorporated into the Turkish analytical dataset.

### 2.3. Data Structure

The evidence base was organized into seven linked but non-interchangeable layers (Table 1). These layers separate the eligible reports retrieved through database and citation searching, the sources used to reconcile the national species total, the taxon-record compilation, the primary and all-explicit province datasets, the vector-evidence layer, and the cross-border contextual layer. Each layer has a distinct analytical unit and supports a defined level of inference; counts from different layers were therefore not treated as alternative estimates of the same quantity.

Within the taxon-record layer, each row represents a species-by-locality, species-by-province, or species-by-habitat record according to the resolution available in the source; when several resolutions were available, the finest extractable geographical unit was retained. Historical occurrence records recovered through secondary syntheses were reassigned, where possible, to the cited original report(s) in the provenance layer; secondary syntheses were retained only as verification or citation-tracing sources. Multi-source occurrences were counted once as occurrence records while retaining all original-report links in a separate provenance crosswalk (Supplementary File S1, Sheets S25 and S26). Eligibility for the evidence corpus was treated separately from analytical inclusion: an eligible historical report was not automatically promoted into the standardized province-level matrices unless it met the analytical data-layer criteria. The Konya breeding-site dataset [23] was classified as secondary because it was based on targeted substrate sampling and laboratory emergence rather than adult-trap surveillance, and it was therefore excluded from the primary province-richness analysis. Pathogen detection, outbreak context, serology, and host-feeding records were retained in the vector-evidence layer and were not used to calculate province-level richness.

Study-design heterogeneity was audited across collection method, sampling period, geographical resolution, specimen or pool denominator, abundance reporting, and identification approach. The audit covered ten principal analytical sources plus one historical national review retained only for verification and citation tracing. The analytical sources included regional adult light-trap surveys using different trap systems, targeted breeding-site sampling, outbreak-associated pooled pathogen screening, molecular identification in selected studies, and a multi-province cattle-farm blood-meal survey. Trap nights, visit frequency, sampling duration, seasonal coverage, habitat coverage, and molecular confirmation were not reported consistently enough to estimate method-specific detection rates or include trapping method as a comparable model covariate. Study count, locality count, and species-by-locality record count were therefore treated as coarse documentation-effort proxies rather than complete corrections for detectability. The source-by-source audit is provided in Supplementary File S1 (Sheet S24).

### 2.4. Spatial, Biogeographical, and Sampling-Bias Analyses

#### 2.4.1. Province-Level Occurrence, Richness, and Evidence-Mapping Dataset

Province-level analyses were based on the standardized literature-derived dataset provided in Supplementary File S1 (Sheets S9 and S16). Reported richness was calculated as the number of distinct *Culicoides* taxa recorded per province in the compiled evidence base. Because the underlying records were extracted from heterogeneous published studies rather than from a standardized national sampling program, richness values were treated as reported or observed richness, not as estimates of true local diversity. Provinces were assigned to dominant biogeographical regions, with 33 of 81 classified as transitional (Section 2.4.2).

Two complementary spatial datasets were used. The first represented reported province-level richness based on primary records and supported the main richness map, bar chart, and effort-conditioned statistical analyses. The second represented sampling coverage and evidence gaps by distinguishing provinces with primary province-level records, provinces represented only by secondary or historical records, and provinces without extractable province-level records in the compiled dataset. These maps were interpreted as evidence maps rather than ecological niche, abundance, or disease-risk maps because comparable data on abundance, vector competence, host density, climate, and pathogen circulation were not uniformly available across Türkiye.

#### 2.4.2. Assignment of Provinces to Biogeographical Regions

To analyze the dataset within an ecological rather than a purely administrative framework, each province was assigned to one dominant biogeographical region: Euro-Siberian, Mediterranean, or Irano-Turanian. The assignment followed the phytogeographical framework established for Türkiye and subsequent vegetation-geographical treatments of Anatolia [24,25,26]. The Euro-Siberian region comprises the humid Black Sea coastal belt, the North Anatolian mountain system, and humid Thrace–Marmara sectors; the Mediterranean region comprises the Aegean and Mediterranean coastal and sub-Mediterranean sectors; and the Irano-Turanian region comprises the interior Central, Eastern, and Southeastern Anatolian steppe-plateau sectors. Province-level assignments are provided in Supplementary File S1 (Sheet S14).

Because several Turkish provinces lie at the interface of major phytogeographical regions, the classification was implemented as a dominant-region framework rather than a strict ecological boundary. A province was flagged as transitional only where published delimitations place it at the documented contact of two of the three regions; that is, where a province assigned to one dominant region also contains documented elements of a second named region. Under this criterion, 33 of the 81 provinces (41%) are transitional: 17 assigned to the Euro-Siberian region, 9 to the Irano-Turanian, and 7 to the Mediterranean. These provinces are concentrated in Thrace–Marmara, the interior Aegean and Lakes district, north-central Anatolia, and the eastern Mediterranean–southeastern Anatolian interface. Provinces of the Eastern Anatolian plateau whose mixed character reflects altitudinal heterogeneity within the Irano-Turanian region, rather than contact with a second region, were retained as core Irano-Turanian.

This distinction was applied consistently with the treatment of elevation elsewhere in the framework. The published records are not sufficiently standardized at sub-provincial elevation or habitat resolution to support an independent alpine classification; the same limitation therefore cannot be used to assign transitional status. If applied consistently, an altitudinal criterion would flag every high-elevation Irano-Turanian province and leave the region without a core, removing the discriminatory value of the classification. The coarseness of province-level assignment in mountainous terrain is instead treated as a limitation of the evidence base (Section 4.6). Transition status is descriptive: the main statistical analyses used the dominant-region classification throughout to avoid fragmenting an already uneven literature-derived dataset into very small regional subgroups and because transitional character is a property of the regional framework rather than of the records. Transition flags are reported in Supplementary File S1 (Sheet S15) and are available for sensitivity checks.

**Figure 1:**
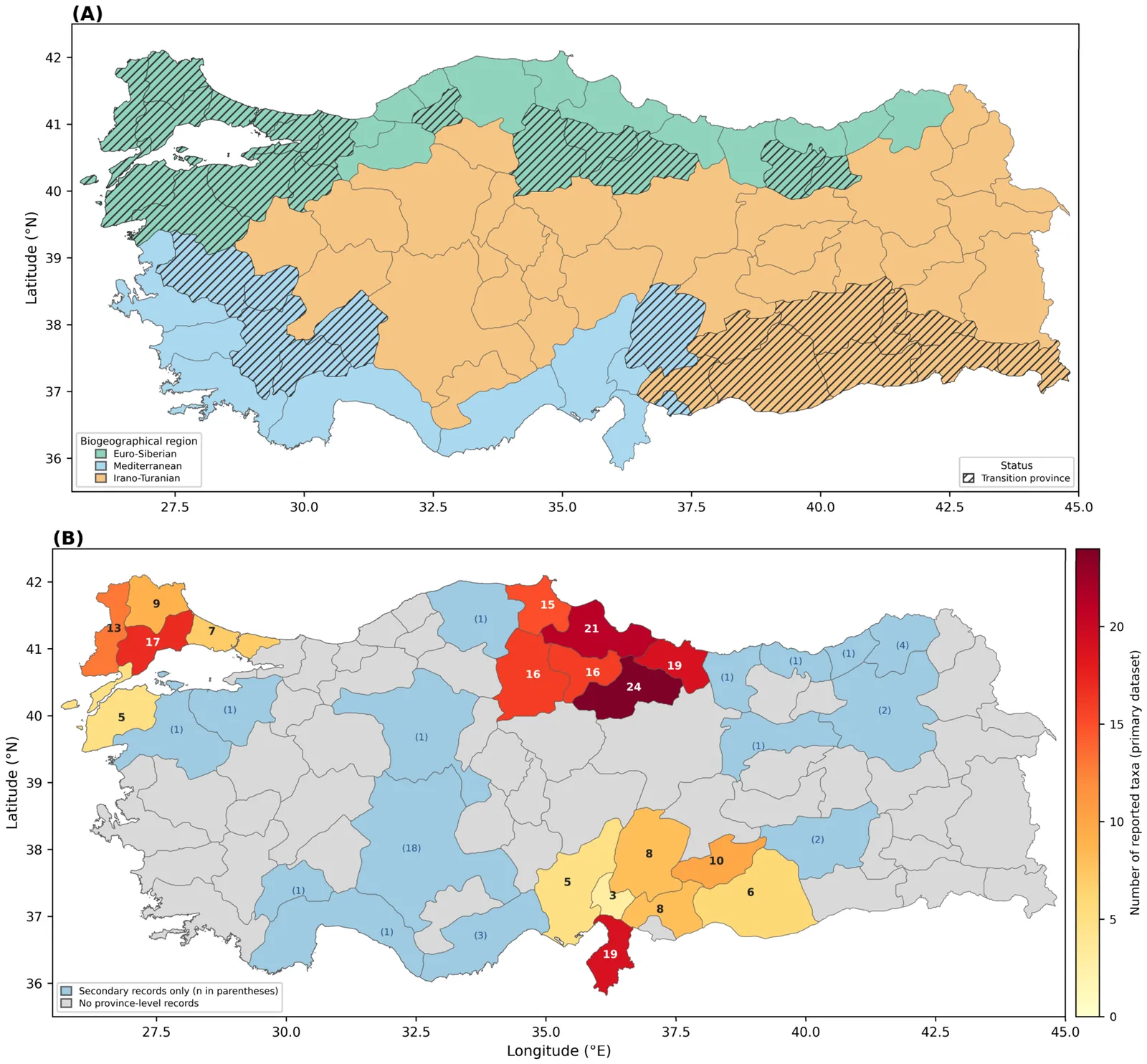
Biogeographical regionalization and spatial coverage of published *Culicoides* records in Türkiye: (**A**) Province-level assignment to the Euro-Siberian, Mediterranean, and Irano-Turanian biogeographical regions. Fill color denotes the dominant biogeographical region, whereas diagonal hatching identifies the 33 provinces classified as transitional under the criterion described in Section 2.4.2. Provinces on the Eastern Anatolian plateau whose mixed character reflects altitudinal rather than inter-regional heterogeneity were retained as core Irano-Turanian. (**B**) Reported *Culicoides* taxon richness and sampling coverage. Provinces supported by primary province-level records are shaded on a sequential richness scale; provinces represented only by secondary or historical records are shown in blue, with reported values in parentheses; provinces without extractable province-level records are shown in light gray. Values are reported (observed) richness from the available literature and are not estimates of true species richness. Both panels use GADM version 4.1 level-1 province boundaries. Regional assignment, transition status, and province-level richness data are provided in Supplementary File S1 (Sheets S9, S14, and S15).

A biogeographical regionalization map was prepared by overlaying dominant province-level regional assignments on the first-level administrative boundaries of Türkiye (Figure 1A). Administrative boundaries were obtained from the Database of Global Administrative Areas (GADM, version 4.1, accessed May 2026) and linked to the province-level classification table using standardized province names. Provinces without extractable occurrence records were retained in the map to show the full spatial extent of the regional framework.

#### 2.4.3. Sampling Effort and Wallacean Shortfall Assessment

Sampling effort was quantified at the province level using three complementary indicators: the number of contributing studies, the number of distinct localities or sampling stations, and the number of species × locality records. The relationship between reported richness and sampling effort was used to evaluate the extent to which the compiled evidence base reflects a Wallacean shortfall, that is, incomplete knowledge of the geographical distribution of species [19]. Spearman rank correlations (ρ) were calculated between reported taxon richness and the main sampling effort variables. Analyses focused on the 18 provinces represented by primary province-level records, because secondary and historical records were not considered sufficient for comparably estimating local sampling effort.

#### 2.4.4. Statistical Modeling of Reported Richness

To evaluate whether apparent regional differences in reported richness persisted after accounting for available documentation effort, province-level richness was modeled as a function of dominant biogeographical region and coarse sampling-effort proxies using generalized linear models [27]. Poisson models used reported taxon richness as the response and included either species-by-locality record count or distinct locality/station count as the effort covariate. The significance of the region term was assessed using likelihood-ratio tests comparing nested models, Wald 95% confidence intervals were obtained for effort coefficients, and overdispersion was evaluated before considering negative binomial alternatives [28]. These models do not remove heterogeneity in trap type, sampling season, duration, habitat coverage, or detection probability; they describe patterns in reported richness after inclusion of the effort information that could be recovered consistently from the literature. Full correlation and generalized linear model outputs are provided in Supplementary File S1 (Sheet S17).

Modeling vector-related evidence as a province-level binomial response was considered but was not defensible. The four principal pathogen-screening or blood-meal studies did not provide comparable province-resolved screened/unscreened denominators, and provinces that were never screened could not be distinguished from provinces screened with negative results. A model would therefore predict where evidence had been published rather than where infection or transmission occurred. The presence of at least one vector-associated taxon was also invariant across the 18 provinces in the primary province dataset. Vector-related findings were consequently synthesized through an evidence-gap framework rather than subjected to regional risk modeling.

Because provinces could be represented by more than one study and individual studies could contribute records from multiple provinces, a single study identity could not be assigned at the province level. To assess whether within-study non-independence affected the conclusions, the analysis was repeated at the study × province level (25 observations from 7 studies). The number of taxa reported by a given study in a given province was the response; dominant biogeographical region and log-transformed sampling effort were fixed effects, and study identity was a random intercept. The model was fitted as a Poisson generalized linear mixed model [29] using a variational Bayes approximation. Full model output is provided in Supplementary File S1 (Sheet S22).

Sensitivity analyses assessed the influence of the main analytical choices on the conclusions. Three scenarios were examined in addition to the two dataset definitions: exclusion of Konya, the most intensively sampled province and the only one whose records derive substantially from targeted breeding-site sampling; the choice between the primary province dataset (18 provinces) and the all-explicit province dataset (33 provinces); and exclusion of the provinces classified as transitional (Section 2.4.2). Each scenario repeated the Spearman correlations and the Poisson generalized linear model with a likelihood-ratio test for the region term. The results are reported in Supplementary File S1 (Sheet S21).

#### 2.4.5. Assemblage Composition and Incidence-Based Richness Analyses

An exploratory province × species incidence matrix was constructed for the primary province dataset to assess whether the reported assemblage structure differed among dominant biogeographical regions. Presence/absence data were used because abundance, trap nights, sampling duration, and detection probability were not consistently available. Pairwise Jaccard dissimilarities were calculated [30], differences in reported composition were tested using permutational multivariate analysis of variance (PERMANOVA) with 999 permutations [31], and principal coordinates analysis (PCoA) was used for visualization [32]. These analyses describe source-conditioned compositional structure; they do not identify climatic, elevational, habitat, livestock-density, or other environmental drivers. Such covariates were not added because they were unavailable at the same spatial and temporal resolution as the heterogeneous occurrence records. To assess whether the PERMANOVA results could be influenced by unequal within-region dispersion, homogeneity of multivariate dispersion was evaluated using permutational analysis of multivariate dispersions (PERMDISP) on the same Jaccard distance matrices [33,34]. Distances to group spatial medians in the full principal-coordinate space were tested with 9999 unrestricted residual permutations using a fixed random seed (20260806). Because regional sample sizes were small and unequal (11/4/3 in the primary province dataset and 18/7/8 in the all-explicit province dataset), the small-sample correction √[n/(n − 1)] was applied to the primary diagnostic; unadjusted results were retained as a sensitivity check; full diagnostics are provided in Supplementary File S1 (Sheet S18), and the underlying primary province × species incidence matrix is provided in Sheet S19.

Total Jaccard dissimilarity (βJAC) was partitioned into the Jaccard turnover component (βJTU; species replacement) and the Jaccard nestedness-resultant component (βJNE; richness-associated subset structure) using the Jaccard-family formulation [35,36]. Partitioning was applied to the primary incidence matrix as both multiple-site and pairwise measures and was summarized separately for within-region and between-region province pairs. Because βJNE increases when the reported assemblage of one province is a subset of a richer province, its association with the absolute difference in reported richness was examined using Spearman’s rank correlation. The components were interpreted as descriptive properties of the literature-derived incidence matrix, not as evidence of ecological mechanism. Full turnover/nestedness partitioning results are provided in Supplementary File S1 (Sheet S23).

Incidence-based richness estimators were used as exploratory diagnostics of undocumented diversity rather than as estimates of the definitive Turkish fauna. Chao2 [37], first- and second-order jackknife estimators [38,39], and the incidence-based coverage estimator (ICE) [40] were applied to the province × species incidence matrix. The Chao2 confidence interval was calculated using the log-normal method [37], and jackknife variance followed [41]. Chao2 is particularly sensitive to taxa recorded in exactly one or two sampling units; in the primary matrix only three taxa occurred in exactly two provinces, so its wide interval was treated as evidence of estimator instability and limited applicability to this heterogeneous literature-derived dataset. Coverage-standardized rarefaction among regions [42] was considered but not applied because the groups contained only 3–11 non-exchangeable province units and produced unstable incidence-frequency profiles. Neither the generalized linear models nor these estimators were treated as a substitute for standardized rarefaction or replicated detection data. ICE sensitivity was examined across infrequent-species cutoffs from five to seventeen sampling units.

### 2.5. Evidence-Gap Interpretation of Vector Relevance

Vector relevance was interpreted through an evidence-gap framework that separates the following: (i) pathogen nucleic-acid detection in field-collected *Culicoides* pools; (ii) negative pathogen screening under specified survey conditions; (iii) host blood-meal evidence; (iv) molecular support for taxonomic identification; and (v) epidemiological or serological context in vertebrate hosts. For each domain, the synthesis distinguished the inference directly supported by the published evidence from stronger inferences that remained unsupported and identified the data required to close that gap. Field detection in whole insects was not equated with biological vector competence, negative screening was not treated as proof of absence, and cattle-farm blood-meal results were not generalized to unrestricted host preference [1,2]. The supporting vector, pathogen, and host-feeding evidence dataset is provided in Supplementary File S1 (Sheet S6).

### 2.6. AI-Assisted Tools and Verification

The AI-assisted tools Claude (Opus 4.8, Anthropic, accessed August 2026) and Codex (OpenAI, accessed August 2026) were used after study selection for language refinement, assistance with script drafting, consistency checking, and recalculation of summary statistics. They were not used to conduct database searches, screen records, determine eligibility, extract source data autonomously, make taxonomic decisions, assign vector status, select statistical conclusions, or generate unverified references. The authors executed the scripts and checked all inputs and outputs against Supplementary File S1 and the cited publications. Maps, figures, taxon counts, province and region assignments, statistical outputs, table entries, captions, interpretations, and conclusions were reviewed and approved by the authors before inclusion. Scripts are available from the corresponding author upon reasonable request.

## 3. Results

### 3.1. Historical Development of Reported Culicoides Richness in Türkiye

Table 2 summarizes the taxonomic progression from the first comprehensive national synthesis through the updated checklist, subsequent new country record, and later monograph (Supplementary File S1, Sheets S3, S4, and S11). The updated checklist and subsequent new-record evidence establish a working total of 72 species [10,11], while the earlier historical review [9] provides taxonomic and distributional context for that reconciliation. The later national monograph [12], retained separately for taxonomic reconciliation, recognizes *C. indistinctus* and *C. vitreipennis* through retrospective taxonomic reassessment and yields a contextual total of 74. The difference is methodological rather than contradictory: 72 represents the working total supported within the eligible evidence chain, whereas 74 represents the later national taxonomic interpretation. Neither value is expected to equal the total in the taxon-record compilation or the province incidence matrices.

### 3.2. Composition of the Analytical Dataset

The taxon-record compilation contained 1164 records extracted at locality, province, or habitat resolution, representing 62 taxa at species or species-complex level. The eligible evidence-report corpus comprised 58 reports consolidated into 43 operational study/data-source families for provenance control. The all-explicit province dataset contained 33 provinces and 45 taxa, while only 18 provinces contributed to the primary province dataset used for the main effort, assemblage, β-diversity, and richness-diagnostic analyses; that dataset contained 43 taxa. The 2026 molecular-evidence record concerned an Aydın *Culicoides imicola* specimen already represented at the province × species level elsewhere in the occurrence corpus; consequently, the incidence matrices in Supplementary File S1 (Sheets S9 and S16) and all province-level analytical results were unchanged. These analytical counts differ from the 72- and 74-species national taxonomic totals because the layers have different source requirements and analytical purposes (Table 1).

The ten principal analytical sources summarized in the study-design audit were not methodologically uniform. They included regional adult light-trap surveys, targeted breeding-site sampling, outbreak-associated pathogen surveys using pooled *Culicoides*, molecular identification in selected studies, and cattle-farm blood-meal sampling; the historical national review used only for verification and citation tracing was documented separately. Trap systems, sampling seasons, duration, spatial coverage, specimen totals, pool construction, and identification procedures varied, and trap-night or visit-level denominators were frequently unavailable. All diversity values are therefore reported or observed richness, and the available effort variables index documentation intensity rather than standardizing species detection. Sampling periods, collection methods, spatial units, sample totals, denominator availability, identification approaches, analytical roles, and comparability limitations are summarized source-by-source in Supplementary File S1 (Sheet S24).

### 3.3. Province- and Region-Level Reported Species Richness

**Figure 2:**
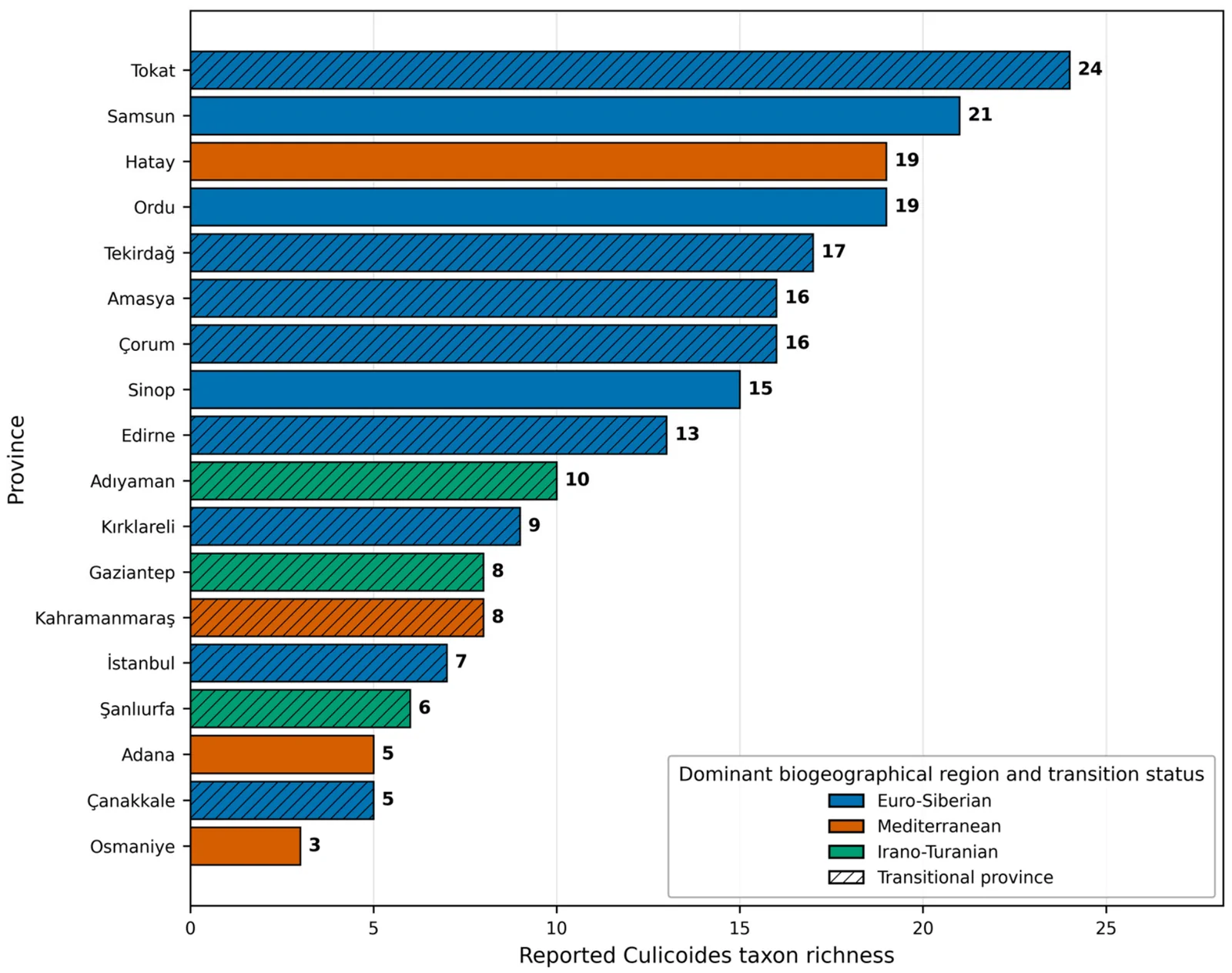
Reported *Culicoides* taxon richness across the 18 provinces in the primary province dataset, ranked from highest to lowest. Bar color denotes the dominant biogeographical region (Euro-Siberian, Mediterranean, or Irano-Turanian), and diagonal hatching identifies the 12 provinces classified as transitional among the 18 provinces in this dataset (of the 33 transitional provinces nationally; Figure 1 and Section 2.4.2). Numbers at the ends of the bars show observed taxon richness. Secondary habitat-based and historical records are excluded; the displayed values therefore summarize the primary published evidence and should not be interpreted as estimates of true provincial richness.

Province-level reported richness remained highly uneven across Türkiye. In the primary province dataset, Tokat had the highest reported richness (24 taxa), followed by Samsun (21) and Ordu and Hatay (19 each). Tekirdağ had 17 taxa, Amasya and Çorum had 16 each, and Sinop had 15. Among the remaining primary provinces, Edirne contributed 13 taxa, Adıyaman had 10, Kırklareli had nine, Gaziantep and Kahramanmaraş had eight each, İstanbul had seven, Şanlıurfa had six, Adana and Çanakkale had five each, and Osmaniye had three (Figure 1B and Figure 2). Konya contributed 18 taxa through a targeted breeding-site study and was therefore retained as a secondary ecological dataset rather than as a primary province-level faunistic record.

When records were interpreted within the dominant biogeographical framework, the Euro-Siberian region showed the highest reported richness and the largest number of contributing provinces. However, this pattern coincided with the strongest concentration of published sampling in the Black Sea and Marmara/Thrace sectors. Mediterranean records were concentrated mainly in the eastern Mediterranean and southern provinces, whereas Irano-Turanian records were comparatively sparse and strongly affected by the exclusion of Konya from the primary province dataset. These regional values therefore reflect the published evidence base rather than true biogeographical richness gradients. The marked sensitivity of Irano-Turanian richness to the inclusion of Konya further indicates that the apparent regional pattern is strongly dependent on geographically concentrated sampling.

### 3.4. Sampling Coverage and Evidence Gaps

Sampling coverage was highly uneven across Türkiye, with only 18 of the 33 provinces in the all-explicit province dataset contributing to the primary analyses (Figure 1B). This pattern represents a clear Wallacean shortfall: the known geographical distribution of Turkish *Culicoides* taxa is incomplete and spatially biased. Provinces with low or no reported richness should therefore not be interpreted as genuinely species-poor, because low richness may reflect limited sampling effort, historical publication gaps, or the absence of extractable province-level data.

### 3.5. Sampling Effort and Reported Species Richness

**Figure 3:**
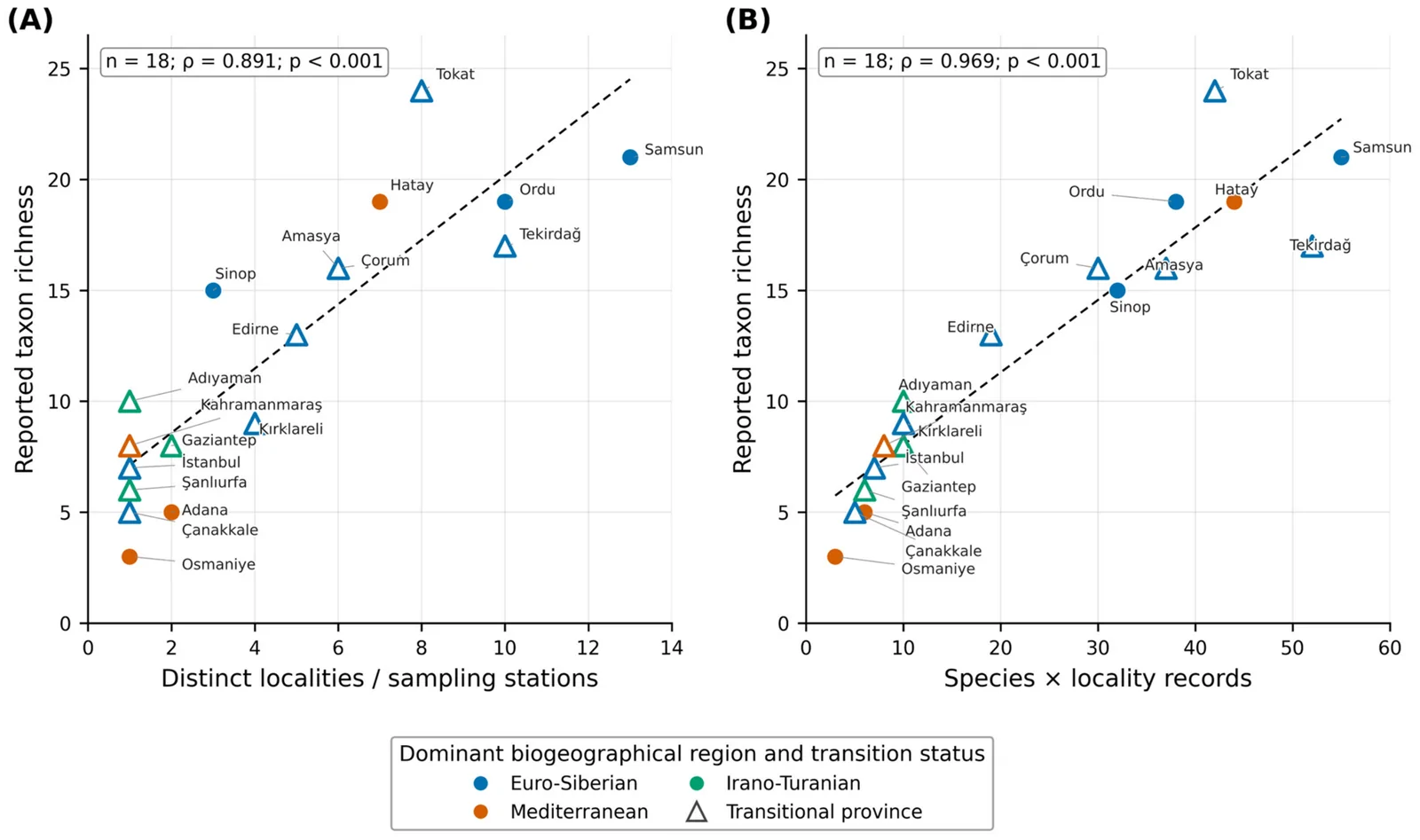
Relationship between recoverable sampling-effort proxies and reported taxon richness across the 18 provinces in the primary province dataset: (**A**) Number of distinct localities or sampling stations (Spearman ρ = 0.891, *p* < 0.001). (**B**) Number of species × locality records (Spearman ρ = 0.969, *p* < 0.001). Point color denotes the dominant biogeographical region. Open triangles identify the 12 transitional provinces, whereas filled circles identify non-transition provinces. Dashed lines are fitted linear trends shown only to aid visualization. The strong associations indicate documentation bias and do not estimate true species diversity.

Spearman rank correlations showed that reported species richness was strongly associated with the recoverable sampling-effort proxies across the 18 provinces in the primary province dataset [9,10,13,14,15,16,43,44,45,46,47]. Reported taxon richness correlated with distinct locality or station count (ρ = 0.891, *p* < 0.001) and even more strongly with species × locality record count (ρ = 0.969, *p* < 0.001; Figure 3). The same pattern occurred in the all-explicit province dataset (33 provinces). In Poisson generalized linear models, log-transformed species × locality record count was significant (coefficient = 0.579, 95% CI: 0.385–0.773, *p* < 0.001), whereas the region term was not significant after inclusion of this proxy (likelihood-ratio χ^2^ = 0.179, df = 2, *p* = 0.914). No overdispersion was detected. These results show that reported richness is strongly conditioned by documentation effort; they do not imply that the model corrected unmeasured differences in trapping method, duration, seasonal coverage, or detectability.

Accounting for study identity did not alter these conclusions. In a Poisson generalized linear mixed model fitted at the study × province level (25 observations, seven studies), the study-level random-intercept standard deviation was 0.140 on the log scale (95% CI: 0.077–0.255), corresponding to approximately ±15% multiplicative variation in reported richness among studies. For comparison, a doubling of sampling effort corresponds to a 1.59-fold increase in reported richness. The effort coefficient was essentially unchanged from the model that ignored study identity (0.667 versus 0.649), and the region terms remained indistinguishable from zero (Mediterranean +0.068, posterior SD 0.135; Irano-Turanian +0.144, posterior SD 0.187). With only seven random-effect groups, the variance component is imprecisely estimated; the key result is the stability of the fixed effects across formulations, suggesting that the strong association with sampling effort is not explained solely by study-level clustering.

The main conclusion was stable across the analytical scenarios reported in Supplementary File S1 (Sheet S21). Excluding Konya, changing the province dataset, and excluding transition provinces did not remove the strong effort-richness association, and the region term was never significant after inclusion of the available effort proxy (likelihood-ratio *p* = 0.387–0.914). However, all three Irano-Turanian provinces in the primary province dataset were transitional, and removing transitional provinces left only six provinces from two regions. This reduced dataset was inadequate for regional testing and illustrates that sensitivity analysis cannot compensate for missing core Irano-Turanian sampling.

Direct comparison of the two province datasets yielded the same substantive inference. In the primary province dataset (18 provinces), the species × locality effort coefficient was 0.579 (95% CI: 0.385–0.773) and the adjusted region term was not significant (likelihood-ratio *p* = 0.914); in the all-explicit province dataset (33 provinces), the corresponding coefficient was 0.667 (95% CI: 0.561–0.774) and the region term again was not significant (*p* = 0.795). The effort–richness correlations based on species × locality records were also strong in both datasets (ρ = 0.969 and 0.990, respectively). Sample coverage was similar (94.3% and 95.1%), while the Chao2 estimates retained wide, overlapping confidence intervals (69.6, 95% CI: 49.5–152.0; and 61.4, 95% CI: 49.4–106.1). Thus, broadening the dataset increased geographical coverage but did not alter the conclusion that recoverable documentation effort remained the strongest predictor of reported richness in the fitted models, as detailed in Supplementary File S1 (Sheets S20 and S21).

### 3.6. Exploratory Assemblage Composition and Incidence-Based Richness Diagnostics

The literature-derived incidence matrices showed a compositional pattern consistent with broad regional differentiation. One-way PERMANOVA based on Jaccard dissimilarity was significant in the primary province dataset (18 provinces; F = 4.388, R^2^ = 0.369, *p* = 0.001; 999 permutations) and in the all-explicit province dataset (33 provinces; F = 2.562, R^2^ = 0.146, *p* = 0.003). These results show that the compiled presence records are not compositionally identical among dominant-region groups; they do not demonstrate that region itself, climate, altitude, livestock density, or another environmental variable caused the pattern. Uneven sampling, unequal group sizes, transition-province assignments, and source-specific taxonomic coverage remain alternative explanations.

PERMDISP provided no evidence that the dominant biogeographical-region groups differed in multivariate dispersion. With the small-sample correction, the primary province dataset yielded F(2,15) = 1.223 and *p* = 0.325, whereas the all-explicit province dataset yielded F(2,30) = 0.421 and *p* = 0.655 (9999 permutations). The conclusion was unchanged without the correction (primary: F = 2.674, *p* = 0.096; all-explicit province dataset: F = 0.156, *p* = 0.857). Thus, the significant PERMANOVA results were not accompanied by statistically supported dispersion heterogeneity, although the small and unequal primary groups limit the power of this diagnostic.

**Figure 4:**
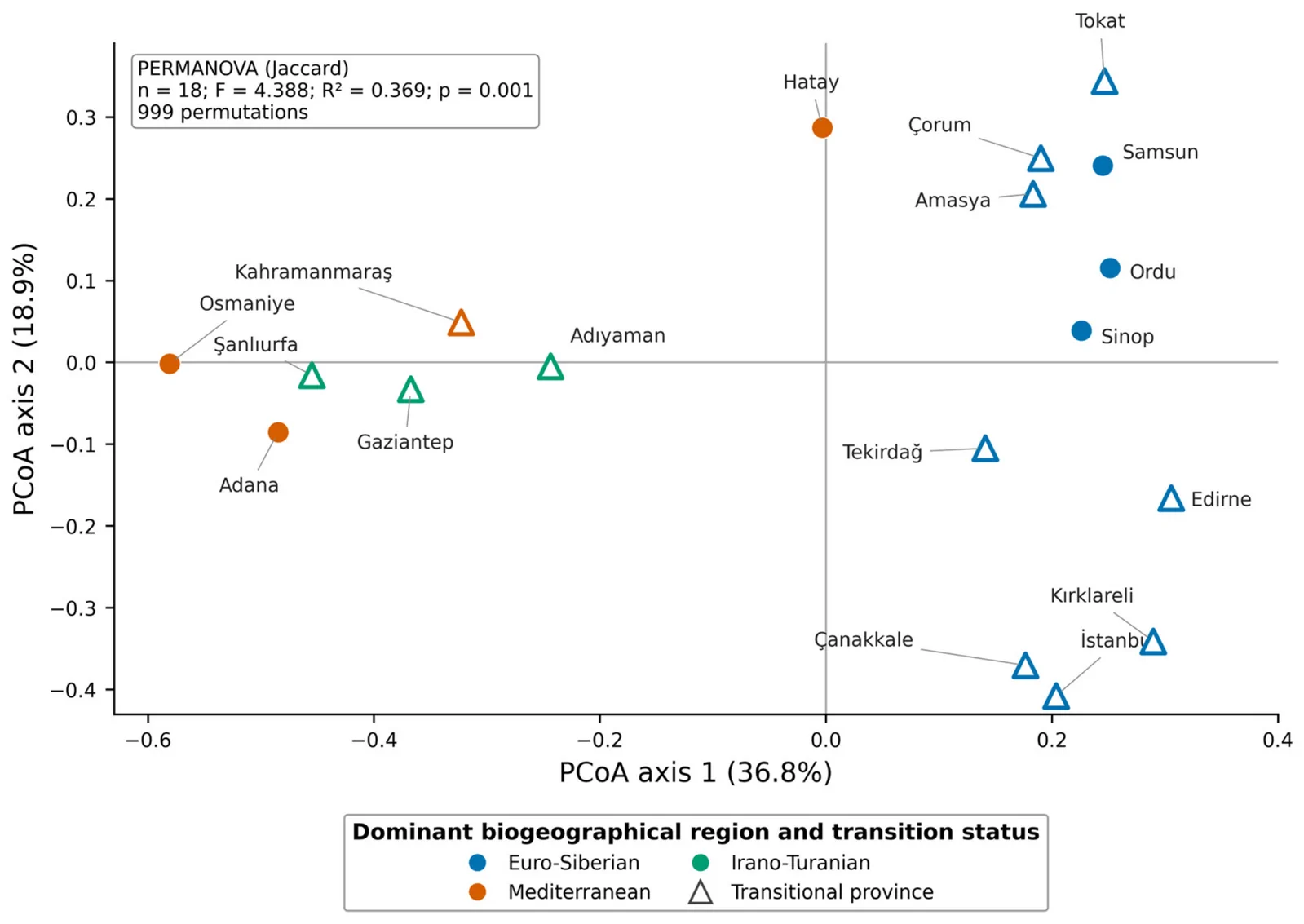
Principal coordinates analysis (PCoA) of province-level *Culicoides* assemblage composition based on Jaccard dissimilarity for the 18 provinces in the primary incidence matrix. Point color denotes the dominant biogeographical region. Open triangles identify the 12 transitional provinces, whereas filled circles identify non-transition provinces. The inset reports the corresponding one-way PERMANOVA result based on 999 permutations. PCoA axes 1 and 2 explain 36.8% and 18.9% of the Jaccard dissimilarity structure, respectively. The ordination summarizes the literature-derived incidence pattern and does not identify its environmental or epidemiological drivers.

PCoA ordination of the primary province dataset summarized this pattern visually (Figure 4). Euro-Siberian provinces clustered mainly on the positive side of the first axis, whereas Mediterranean and Irano-Turanian provinces tended to occupy more negative coordinates. The first two PCoA axes explained 36.8% and 18.9% of the Jaccard dissimilarity structure, respectively. Transition provinces (the 12 transitional provinces within the primary province dataset) did not, however, occupy systematically intermediate positions: their mean absolute first-axis score (0.26) was close to that of non-transition provinces (0.30), and the most centrally placed province on the first axis was Hatay, classified as core Mediterranean. Transition status therefore appears to be a property of the regional framework rather than a compositional gradient resolvable from the present records (Section 2.4.2), consistent with the dominant-region classification being an analytical simplification rather than a sharp ecological boundary.

For the primary province dataset (18 provinces, 43 observed taxa), first-order jackknife estimated 55.3 taxa (SE 4.5; 95% CI: 46.4–64.1), second-order jackknife 64.3, and ICE 54.0; ICE remained stable across cutoffs from five to seventeen units (54.0–57.3). Chao2 produced 69.6 taxa with a much wider interval (SE 21.9; 95% CI: 49.5–152.0). Its instability is expected because the estimator is highly sensitive to the ratio of taxa occurring in one versus exactly two sampling units, and only three taxa occurred in exactly two provinces. For the all-explicit province dataset (33 provinces), Chao2 remained uncertain (95% CI: 49.4–106.1). The clustering of ICE and the two jackknife diagnostics supports the limited conclusion that additional taxa are likely to be documented, whereas the Chao2 interval demonstrates that the magnitude cannot be estimated reliably from these heterogeneous province units [48]. None of the estimators was used to revise the 72- or 74-species national taxonomic total.

Coverage-standardized rarefaction across biogeographical regions was not applied. The primary province dataset contained 11 Euro-Siberian, four Mediterranean, and three Irano-Turanian province units, which were neither balanced nor exchangeable in method or intensity. The apparently high coverage of the sparsely represented Irano-Turanian group arose from its incidence-frequency structure and did not demonstrate adequate inventory completeness. Regional comparisons were therefore restricted to effort-conditioned richness models and exploratory presence/absence analyses, while explicitly recognizing that neither provides independent coverage-standardized evidence.

Partitioning of Jaccard dissimilarity showed that turnover accounted for 91.3% of multiple-site dissimilarity across the 18 provinces in the primary province dataset and approximately 78% of mean between-region dissimilarity. Between-region pairs were more dissimilar and more turnover-dominated than within-region pairs. This pattern is consistent with broad differentiation in the reported species lists, but it remains a property of the compiled incidence matrix and does not reveal the environmental process producing replacement.

Within-region comparisons exposed the stronger influence of sampling structure. The four Mediterranean province lists formed a perfectly nested sequence, with no recorded replacement among progressively poorer lists, and pairwise βJNE was strongly associated with absolute richness difference (ρ = 0.628, *p* < 0.001), whereas βJTU was not. Because reported richness itself tracks documentation effort, Mediterranean nestedness is more plausibly interpreted as progressive representation of a shared recorded pool than as a demonstrated ecological mechanism. Standardized sampling is required to test whether between-region turnover persists after equalizing method, season, habitat, and effort.

### 3.7. Publication Trends in Culicoides Research in Türkiye

**Figure 5:**
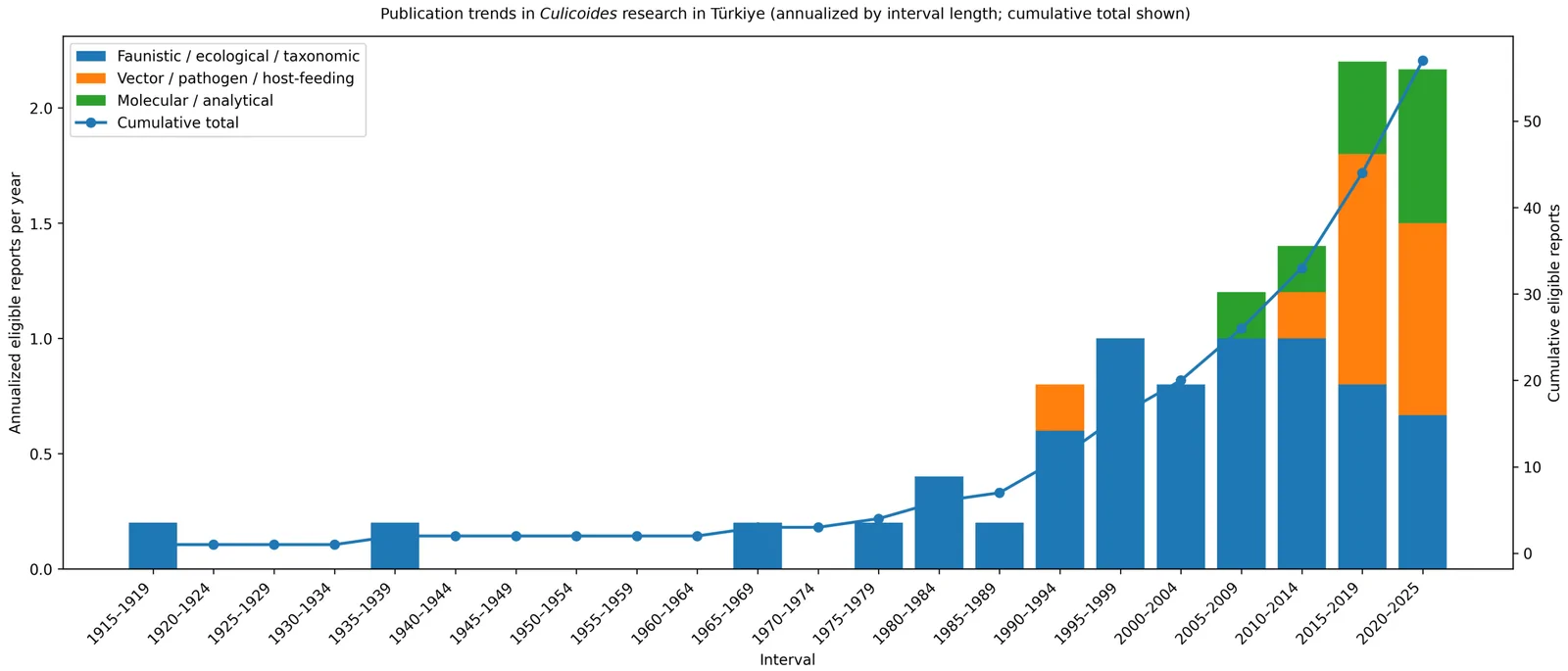
Publication trends in *Culicoides* research in Türkiye based on the completed-year temporal subset of 57 eligible reports published between 1918 and 2025. Stacked bars show annualized report counts (reports per year) within calendar intervals, grouped as faunistic/ecological/taxonomic, vector/pathogen/host-feeding, and molecular/analytical. Intervals are 5-year blocks except the terminal interval (2020–2025), which spans six years; all bar heights are normalized by interval duration so that the terminal period is directly comparable with the preceding blocks and does not create an artificial decline caused solely by unequal interval length. The line on the right-hand axis shows raw cumulative eligible-report counts, reaching 57 in 2025. One additional eligible report was published in 2026, bringing the complete evidence corpus to 58 reports; it is not included in this temporal-rate figure because 2026 was incomplete when the literature search was completed in August 2026. Temporal patterns describe the assembled evidence corpus rather than standardized research effort.

The complete eligible evidence corpus contains 58 reports through August 2026, whereas the completed-year temporal subset used for Figure 5 contains 57 reports published between 1918 and 2025. Publication activity was sparse before 1980 and increased thereafter. On an annualized basis, output was highest in 2015–2019 (2.20 eligible reports/year) and remained similar in 2020–2025 (2.17 reports/year), rather than showing a clear terminal decline (Figure 5). Faunistic/ecological/taxonomic reports dominate the earlier corpus, whereas vector/pathogen/host-feeding and molecular/analytical reports become more prominent in recent intervals. The cumulative completed-year total reaches 57 reports by 2025. One additional eligible report was published in 2026 and is included in the complete evidence corpus but not in the temporal rate because 2026 was incomplete at the August 2026 search date. These counts describe publication activity only and are not standardized measures of research effort or ecological or epidemiological change.

Within the completed-year subset, the temporal profile provides additional context for the Wallacean shortfall evident in the spatial analyses. Only four of the 57 eligible reports published through 2025 (7%) predate 1980, whereas 41 (72%) were published from 2000 onward and 31 (54%) from 2010 onward. The province-level studies contributing to the primary incidence matrix are also predominantly recent. Thus, the evidence base is temporally concentrated as well as spatially uneven: much of the currently available literature accumulated during the last two to three decades rather than through a long-term, spatially systematic national survey. The increase in publication activity also coincides with the expansion of molecular approaches and growing surveillance interest in *Culicoides*-borne pathogens, although the present data do not allow these factors to be tested as causes of the publication pattern. This temporal concentration helps explain why reported richness remains strongly conditioned by where, when, and how intensively the fauna has been examined rather than directly representing its underlying biological distribution.

### 3.8. Geographic Range Breadth of Recorded Culicoides Species

**Figure 6:**
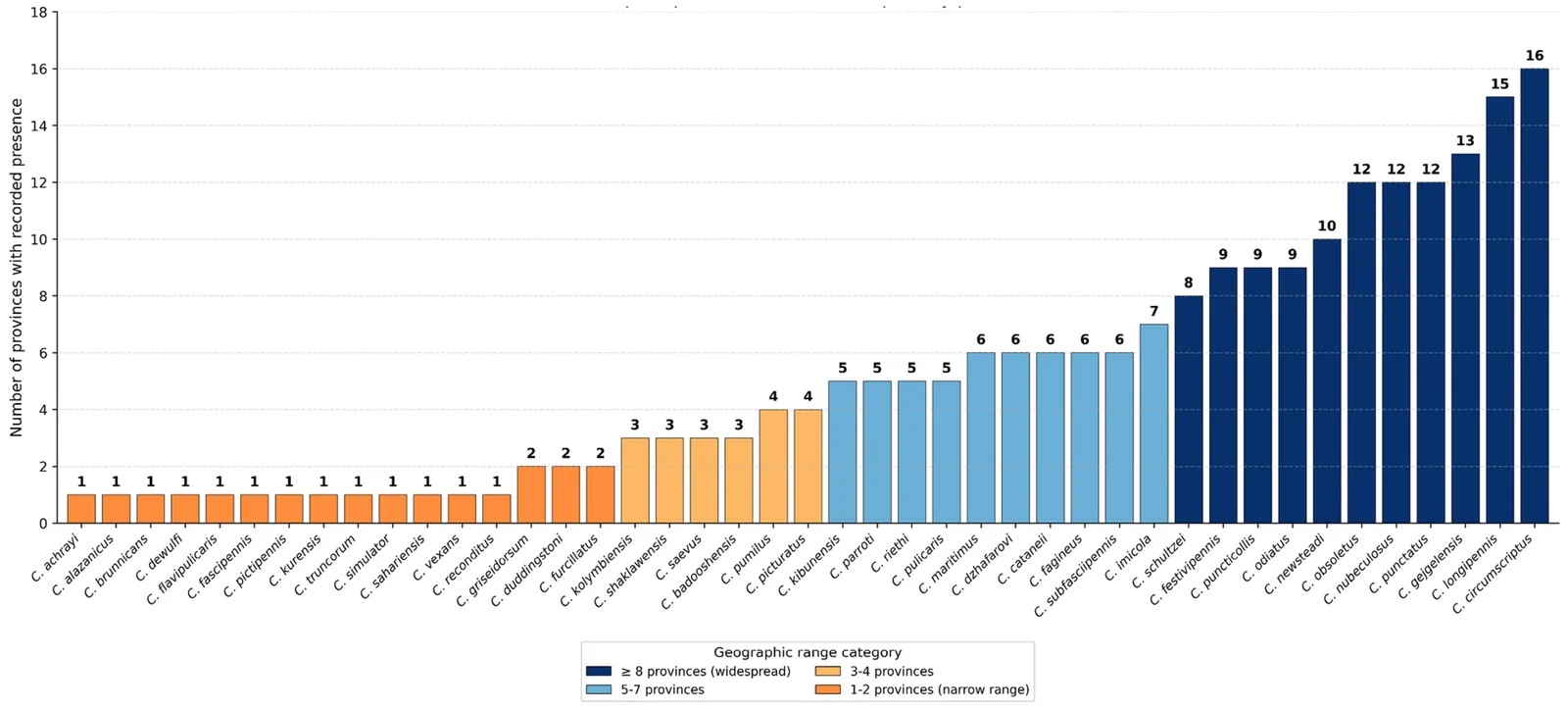
Reported geographic range breadth (number of provinces with recorded presence) for 43 *Culicoides* taxa in Türkiye, based on the primary province-level occurrence dataset. Bars are categorized by range breadth: widespread (at least 8 provinces, dark blue), intermediate (5–7 provinces, light blue), restricted (3–4 provinces, yellow), and narrow range (1–2 provinces, orange). Values reflect reported occurrences from available published records only and should not be interpreted as true range-size estimates.

Province-level occurrence data in the primary province dataset were available for 43 *Culicoides* taxa. Of these, *C. circumscriptus* and *C. longipennis* had the broadest documented geographic ranges, with records from 16 and 15 provinces, respectively (Figure 6). Eleven species were recorded from eight or more provinces and may be considered widespread within the published record, whereas 16 species (37%) were documented from only one or two provinces. These apparently narrow ranges should be interpreted cautiously because they may represent under-sampling outside targeted study areas rather than true range restriction. The median documented range breadth was four provinces per species, reflecting the geographically constrained nature of the compiled dataset [9,10,13,14,15,16,43,44,45,46,47]. The strongly right-skewed range-breadth distribution, with many taxa recorded from only one or a few provinces and relatively few recorded widely, is consistent with the geographically uneven and incomplete coverage of the compiled evidence.

### 3.9. Vector-Related Evidence Retained for Epidemiological Interpretation

The principal arbovirus-related evidence comprised AKAV, SBV/BTV, and BEFV investigations. Seven AKAV-positive pools were reported from the Eastern Mediterranean: five involving the *C. schultzei* complex, one *C. longipennis*, and one *C. circumscriptus* [7]. A separate 2026 molecular study generated the complete 702 nt AKAV N-gene sequence from a previously AKAV-positive *Culicoides imicola* specimen collected in Aydın in 2017 [49]. This provides additional molecular epidemiological characterization of Turkish *Culicoides*-associated AKAV material but does not represent an independent field-detection event or establish vector competence. In Tekirdağ, SBV RNA was detected in one pool containing a single unengorged *C. festivipennis* female, whereas BTV was not detected in the tested material [16]. During the bovine ephemeral fever (BEF) outbreak-associated survey, BEFV was detected in 13 of 35 cattle blood samples but not in 20,845 *Culicoides* specimens grouped into 155 groups [15]. These findings document field association, molecular characterization, local virus circulation, or non-detection under specified survey conditions; none provides comparative infection prevalence or experimental vector competence. Beyond arboviruses, a bovine leukemia virus genome was detected in a *C. schultzei* pool, and molecular screening in İzmir reported *Culicoides* taxa with putative vector potential for haemosporidian parasites; both were treated as exploratory pathogen-association records rather than evidence of established vector roles [50,51]. Table 3 retains the study-level evidence, while the corresponding inference gaps are summarized later in the evidence-gap framework. Additional field studies addressed PPRV detection in *C. imicola* and broader arbovirus screening of hematophagous mosquitoes and midges in Türkiye [52,53]; these reports were retained in the surveillance evidence corpus but were not treated as demonstrations of vector competence.

Using a broad literature-derived vector-association category [1,2], all 18 provinces in the primary province dataset contained at least one taxon assigned to that category. Because this response was invariant and depended on a broad external status category, it could not support regional probability modeling or a ranking of transmission potential. Its defensible implication is limited to surveillance coverage: the widespread presence of broadly vector-associated taxa across the sampled provinces does not, by itself, distinguish regional transmission potential.

Host-feeding evidence was derived from 1225 engorged females grouped into 69 pools from 24 cattle farms in 20 provinces [17]. Cattle blood was detected in all pools, whereas the other tested hosts were negative. The result supports cattle feeding within the cattle-farm sampling frame but cannot distinguish unrestricted host preference among regions because trap placement and host availability were not balanced. Serological and outbreak reports provide epidemiological context but were kept separate from entomological pathogen detection.

Field pathogen detection, negative screening, host-feeding evidence, and vertebrate-host epidemiological context were retained as distinct evidence types. Table 3 documents the original study-level findings, whereas the evidence-gap framework presented below states the inference supported by each domain, the inference that remains unsupported, and the data required for stronger assessment.

### 3.10. Reported Richness and Evidence Across Biogeographical Regions

When province-level records were aggregated into Türkiye’s three principal biogeographical regions, reported richness was uneven (Table 4). The Euro-Siberian region contained 41 reported taxa across 18 contributing provinces, the Mediterranean region 21 taxa across seven provinces, and the Irano-Turanian region 12 taxa across seven provinces when Konya was excluded as a secondary breeding-site dataset. These counts summarize the published evidence layers and are not sampling-standardized estimates of true regional richness. Because *C. salinarius* was recorded only from Konya, 44 of the 45 taxa in the all-explicit province dataset entered this regional comparison. The concentration of apparently region-exclusive taxa in the Euro-Siberian sector parallels its greater province representation and documentation intensity; this pattern is consistent with sampling-conditioned regional structure and should not be interpreted as evidence of regional endemicity. The corresponding regional summary and recalculated Table 4 values are provided in Supplementary File S1 (Sheet S13).

The vector evidence is more informative when arranged by inferential domain than when reduced to regional presence/absence symbols. Table 5 therefore summarizes the available Turkish evidence, the conclusion directly supported, the stronger conclusion that cannot be drawn, and the critical data gap. Positive field-detection evidence consists of seven AKAV pools and one SBV pool; a separate 2026 study generated complete N-gene molecular characterization from one previously AKAV-positive Aydın *C. imicola* specimen [49]. BTV and BEFV were not detected in the tested *Culicoides* material, and host-feeding evidence derives from one cattle-farm survey. The absence of harmonized trap-night, abundance, infection-prevalence, climate, livestock-density, and host-availability data precludes a species–pathogen correlation matrix, regional relative-risk comparison, or epidemic risk-prediction model.

## 4. Discussion

### 4.1. National Richness and Taxonomic Interpretation

This review documents substantial reported *Culicoides* richness in Türkiye while showing that its apparent spatial distribution is strongly influenced by the structure of the published evidence base. The current checklist and peer-reviewed literature on new country records support 72 reported species from Türkiye [10,11], whereas a subsequent national faunistic monograph recognizes 74 species after retrospective taxonomic reassessment [12]. The later interpretation is considered here within the broader taxonomic context provided by the global biting-midge catalog [54]. The distinction is important: 72 is the working total supported within the eligible evidence chain, whereas 74 reflects a later national taxonomic interpretation that updates the faunistic context. Both values indicate that Türkiye contains a substantial documented component of the Western Palaearctic *Culicoides* fauna, consistent with its position at the intersection of the Euro-Siberian, Mediterranean, and Irano-Turanian systems.

The increase from earlier national syntheses to the present totals should not be interpreted as a linear rise in biological diversity alone; it also reflects the accumulation of targeted faunistic surveys, new country records, improved regional coverage, and continued taxonomic revision. In this sense, the historical trajectory of the national species total is shaped by both documentation and taxonomic interpretation. The distinction between recorded diversity and true diversity is therefore central to interpreting Turkish *Culicoides* records.

### 4.2. Wallacean Shortfall as a Central Pattern in Turkish Culicoides Records

The strongest pattern emerging from the analyses is a clear Wallacean shortfall: knowledge of the geographical distribution of Turkish *Culicoides* species is incomplete, spatially uneven, and strongly shaped by where surveys have been conducted. Provinces with high reported richness, such as Tokat, Samsun, Ordu, and Hatay, are also provinces with relatively intensive published sampling, whereas large areas of the country remain poorly represented or lack structured province-level records. Low reported richness should therefore not be treated as evidence of genuine faunistic impoverishment, and the absence of published records should not be interpreted as species absence.

Quantitative sampling-effort analyses support this interpretation. Reported provincial richness was strongly correlated with the number of distinct localities or sampling stations and even more strongly with species-by-locality record counts (Figure 3). In the effort-conditioned modeling framework, the available sampling-effort proxies were the strongest recoverable predictors of reported richness, whereas the independent effect of biogeographical region was weak after those proxies were included. These results shift interpretation of the dataset from a richness map to an evidence map: the maps and richness summaries show where *Culicoides* diversity has been documented, not necessarily where diversity is greatest.

This interpretation also affects species-level range breadth. Many taxa recorded from only one or two provinces may represent genuinely localized species, but they may equally represent taxa that have not been sought, or not reported, outside the provinces where targeted studies were conducted. The large number of narrow-range records in the compiled dataset should therefore be interpreted as a combination of biological range restriction, uneven sampling, and publication bias rather than as direct evidence of endemicity or strict regional confinement. This pattern yields a clear alternative hypothesis for future work: many apparently narrow-range taxa may either be genuinely localized or simply under-recorded because comparable sampling is lacking across provinces. Standardized multi-province surveys would distinguish these alternatives—substantial range expansion under equalized sampling would support an effort-limitation explanation, whereas persistence of restricted distributions would provide stronger evidence for genuine geographical localization.

### 4.3. Effort-Conditioned Biogeographical Interpretation

The province-level biogeographical framework provides a consistent way to organize the evidence but does not eliminate uneven sampling. The Euro-Siberian sector has the highest reported richness and the most apparently exclusive taxa, while also containing the most intensively documented Black Sea and Thrace provinces. Inclusion of locality or species-by-locality effort proxies did not support an independent regional richness effect, but those proxies cannot standardize trap type, duration, season, habitat coverage, or detection probability. The result is therefore effort-conditioned rather than fully sampling-corrected.

The presence/absence analyses add a second, exploratory level of interpretation. Turnover-dominated between-region dissimilarity (approximately 78% of mean between-region dissimilarity; Section 3.6) is consistent with broad faunal differentiation, whereas within-region nestedness, particularly the perfectly nested Mediterranean lists, closely follows reported richness and is compatible with progressive under-sampling. These patterns generate hypotheses rather than demonstrate mechanisms. This dependence yields a directly testable prediction for future surveys: if the present Irano-Turanian richness pattern is driven primarily by sampling concentration rather than by a uniquely species-rich Konya fauna, standardized sampling across additional Irano-Turanian provinces should reduce the current disparity and recover additional taxa outside Konya. Climate, elevation, habitat, livestock density, abundance, and study period were not available at commensurate resolution, so no defensible driver model could distinguish biogeographical filtering from source structure.

The PERMDISP diagnostic (Section 3.6) reduces, but does not eliminate, concern that the PERMANOVA result reflects unequal within-region spread: no statistically supported dispersion heterogeneity was found in either matrix. Its power is nonetheless limited by the small, unbalanced regional groups, in which differences in multivariate location and dispersion are difficult to separate [33,34]. The regional composition result should therefore remain an exploratory, source-conditioned pattern rather than evidence of a discrete ecological boundary.

Transition provinces provide a particularly useful prospective test because they were identified independently of the *Culicoides* outcomes and lie between the dominant biogeographical sectors rather than forming a fourth analytical region. If assemblage composition is genuinely structured by broad biogeographical filtering, standardized samples from transition provinces should show greater cross-sector species sharing and, in ordination space, more intermediate composition than core provinces sampled with the same method, season, habitat coverage, and effort. If transition status contributes little once those design factors are equalized, the apparent boundary structure would instead be more consistent with documentation and sampling history. This prediction gives the transition-zone classification a falsifiable ecological role without claiming that the present province aggregates already demonstrate an ecotonal effect.

Richness diagnostics likewise support only a bounded conclusion. ICE and the two jackknife estimates clustered between 54 and 64 taxa in the primary incidence matrix, suggesting undocumented taxa within that analytical layer. Chao2 was substantially higher and carried a 49.5–152.0 interval because only three taxa occurred in exactly two provinces. The interval is not merely a generic statement of uncertainty; it shows that Chao2 has limited numerical stability in this heterogeneous literature-derived matrix. These diagnostics should not be used to replace the source-reconciled national totals of 72 and 74.

### 4.4. Türkiye Within Broader Transboundary Biogeographical Systems

Although the analytical dataset is restricted to Türkiye, the biogeographical regions used here extend beyond national borders. The Euro-Siberian component of northern Türkiye connects with southeastern Europe, the Black Sea basin, and the Caucasus; the Mediterranean component links western and southern Türkiye with the Aegean and Levantine systems; and the Irano-Turanian component extends toward the Iranian Plateau and western Asia. Figure 1A should therefore be understood as the Turkish sector of broader transboundary ecological systems rather than as a closed national biogeographical unit. This broader framing is consistent with European analyses showing that *Culicoides* vector assemblage composition varies among Köppen–Geiger climate classes [55] and with syntheses documenting *Culicoides* species composition, ranges, breeding sites, and host associations across Russia and adjacent lands [56].

Comparisons with neighboring and regional faunas were restricted to sources supporting a national or broad regional inventory (Table 6). Localized surveys and disease-focused reports informed the assessment of evidence coverage but were not combined with national totals, avoiding asymmetric comparisons between scattered records and formal checklists. Even among retained inventories, totals remain conditioned by survey recency, spatial coverage, and taxonomic treatment and are therefore used only for contextual interpretation.

The contextual search also showed geographical differences in evidence completeness. Western and Balkan sources include broad surveys and updated checklists [57], whereas eastern and southeastern evidence is older or fragmented. A localized Iranian light-trap study yielded four new national records and discussed a cumulative total of 40 species [58], whereas an earlier country-wide synthesis reported 43 species (Table 6). Because the later study was regionally sampled and applied a different taxonomic reconciliation, Table 6 retains the national-synthesis value rather than treating the two totals as directly comparable. For Georgia, Iraq, and Syria, the searches retrieved disease, host, or other partial records rather than a defensible national *Culicoides* inventory. These gaps are relevant to transboundary planning but do not justify quantitative country ranking.

Where comparable evidence is available across a border, it can reveal biogeographical continuity obscured by national boundaries. In the Aegean sector, the documented sequence of the 1998 bluetongue virus serotype 9 incursion through Rhodes, Kos, Leros, and Samos before reaching the Greek mainland [59] is paralleled by the close molecular relationship among Mediterranean *Culicoides imicola* populations from Anatolian Türkiye, the Aegean islands, and mainland Greece [60]. Together, these lines of evidence are consistent with cross-border connectivity but do not imply that the entire fauna or epidemiological system is homogeneous. Continental *Culicoides* distribution models [61], climatic suitability analyses [62], and phylogeographic studies of bluetongue virus dispersal in Europe [63] likewise show that political boundaries need not correspond to ecological or dispersal boundaries. Conversely, the uneven availability and recency of inventories adjoining eastern Türkiye suggest that documentation gaps may also extend across borders. The contextual search was not designed to quantify a west–east gradient in the Wallacean shortfall, but this asymmetry supports harmonized sampling in poorly documented shared biogeographical sectors. Such surveys could test whether assemblage similarity is greater within shared sectors across national borders than between contrasting sectors within Türkiye.

This transboundary perspective is particularly relevant for vector surveillance. *Culicoides* distributions, host availability, livestock movement, wind-assisted dispersal, and arbovirus circulation operate across ecological corridors rather than political boundaries [63,64]. A recent Middle East synthesis similarly describes geographically fragmented entomological and entomovirological evidence, limited systematic vector monitoring relative to animal-based surveillance, and a need for coordinated longitudinal *Culicoides* sampling, standardized molecular screening, improved taxonomic resolution, and integration of vector and host data [65]. Türkiye’s position between Europe, Asia, and the Middle East therefore makes it an important link for faunistic and surveillance data across the Eastern Mediterranean and adjacent Palaearctic regions. The present Türkiye-focused synthesis provides a structured, provenance-controlled national baseline for broader comparison but is not a complete regional synthesis. Future regional work should use harmonized locality-level records, updated taxonomy, and comparable sampling-effort metrics across Türkiye and neighboring countries.

**Table 6:** Reported *Culicoides* species richness in selected neighboring countries of Türkiye (Greece, Armenia, and Iran) and in other Western Palaearctic and Mediterranean countries for which a national or broad inventory supports a defensible species total, ordered by inventory year.

Country	Inventory Year	Reported Culicoides Species	Source
Armenia	2025	14	[66]
Algeria	2020	59	[67]
Morocco	2019	54	[68]
Ukraine	2017	55	[69]
Greece	2009	39	[59]
Israel	1976	39	[70]
Iran	1974	43	[71]

Note. Entries are restricted to selected neighboring and other Western Palaearctic or Mediterranean countries for which the contextual search identified a national or broad inventory supporting a defensible *Culicoides* total. The selected neighboring countries should not be read as a complete list of Türkiye’s land neighbors. Localized, single-survey, host-focused, and disease-focused studies were assessed for contextual coverage but were not combined with national totals. Reported values are not strictly equivalent because inventory date, taxonomic treatment, sampling method, and spatial effort differ. The table is therefore contextual and does not support formal ranking. For Türkiye, the source-reconciled totals are 72 within the eligible evidence chain and 74 under the later national monograph (Section 3.1).

### 4.5. Vector Evidence as an Evidence-Gap Framework

The Turkish vector literature is too sparse and heterogeneous for a species–pathogen correlation matrix or quantitative ranking of vector risk. Positive field-detection evidence is limited to seven AKAV-positive pools involving three taxa or taxon groups and one SBV-positive pool containing a single *C. festivipennis* female [7,16]. The 2026 analysis of a previously AKAV-positive Aydın *C. imicola* specimen adds complete N-gene molecular characterization, but not another independent field detection [49]. BTV and BEFV were not detected in the tested *Culicoides* material [15,16], and host-feeding evidence derives from one cattle-farm study [17]. Table 5 converts this scarcity into an explicit evidence-gap framework rather than presenting the studies as equivalent lines of vector incrimination.

The framework separates field association from competence, and non-detection from absence. Viral RNA in a whole-insect pool can demonstrate contact or field association but cannot establish dissemination to salivary tissues or biological transmission. A negative pool result applies only to its sampling period, species composition, pool design, and assay sensitivity. Cattle blood in traps operated at cattle farms confirms feeding within that sampling frame but cannot quantify regional host preference. Thus, the current literature indicates where particular evidence types have been generated, not where transmission risk is highest.

Stronger inference requires using the same spatial and seasonal sampling units to provide species-resolved abundance, trap-night denominators, molecular identification, blood meal results, pathogen screening, vertebrate host availability, livestock density, and environmental covariates. The study by Pekmez et al. [49] also illustrates a complementary opportunity: archived diagnostic vector material can yield additional molecular epidemiological information years after collection, provided specimen or sample identifiers and provenance are retained. Linking field collections, diagnostic pools, vouchers, and sequence accessions through stable identifiers would therefore allow future surveillance to connect occurrence, infection, and genomic evidence without treating later reanalysis as a new field event. Only with such integrated data could infection prevalence, species–pathogen–host associations, abundance-weighted vector assemblages, or regional transmission potential be modeled. Until those data exist, the appropriate output is a prioritized evidence-gap assessment and a standardized sampling design rather than a predictive risk surface.

### 4.6. Limitations of the Evidence Base and Analytical Framework

First, the dataset reflects substantial heterogeneity in study design. Source studies differed in adult versus immature sampling, trap system, placement, season, duration, visit frequency, geographical resolution, specimen retention, pool construction, abundance reporting, and morphological or molecular identification. These differences can alter detection rates independently of biological richness. Because trap-night and repeated-visit denominators were not reported consistently, method effects could not be quantified or corrected; locality and record counts remain coarse proxies for documentation.

Second, provinces are coarse and internally heterogeneous ecological units. Assigning a single dominant region was necessary for consistent analysis, but many provinces span climatic, elevational, habitat, and land-use gradients. Climate, altitude, livestock density, host availability, and *Culicoides* abundance were not available at a common locality-by-season resolution. Using province averages would not reconstruct the conditions of the original records and could create an ecological fallacy; mechanism-level or epidemic-risk models were therefore withheld.

Third, the assemblage and richness diagnostics are constrained by non-exchangeable province units and publication bias. PERMANOVA, PCoA, and β-diversity partitioning describe incidence structure but do not identify its drivers. Coverage-standardized regional rarefaction was unstable because regional sample sizes were small and unbalanced. Chao2 was especially sensitive to the small number of taxa occurring in exactly two provinces and produced a confidence interval extending to 152 taxa, indicating limited applicability rather than a precise richness estimate. The more stable estimators support likely under-documentation but do not justify revising the national species total.

Finally, the Türkiye-focused synthesis cannot provide a complete cross-border fauna. The contextual search reviewed national inventories and scattered regional studies, but only the former supported comparative totals. A harmonized transboundary analysis would require shared taxonomic reconciliation, standardized locality identifiers, effort metadata, and consistent inclusion rules across countries. Appending heterogeneous regional records to the Turkish matrix would increase apparent coverage without making the units comparable.

### 4.7. Implications for Future Culicoides Research and Surveillance

A prospective national and transboundary protocol should use a standardized adult-trap model and reporting unit, including trap nights; repeated visits within common seasonal windows; and stratification by biogeographical sector, transition zone, elevation, habitat, and host setting. Morphological identifications should be linked to voucher specimens, with molecular confirmation for difficult taxa. A shared site database should connect location, date, method, abundance, specimen or pool identifier, blood-meal result, pathogen assay, host availability, livestock density, and environmental measurements. Common standard operating procedures and Darwin Core-compatible metadata [72] would allow records from Türkiye and adjacent countries to be integrated without erasing provenance.

Climate change should be treated as a prospective vector–ecology hypothesis rather than inferred from current publication chronology. Long-term standardized evidence from elsewhere in the Palaearctic shows why a simple uniform-warming narrative is inadequate: Sanders et al. [73] analyzed approximately 150,000 *Culicoides* from 2867 collections over nearly four decades at two UK sites and found an approximately 40-day extension of adult activity at one site, but no comparable seasonal shift at the other; the lengthening was associated with local changes in temperature and precipitation. A recent bunyavirus review likewise emphasizes that climatic effects on vector-borne transmission emerge through interacting changes in seasonality, vector ecology, dispersal, hosts, and other environmental conditions rather than temperature alone [74]. For Türkiye, the hypothesis should therefore be region- and transition-specific. Standardized longitudinal sampling should test whether Mediterranean and transition-zone sites show earlier onset or longer adult activity and increased inland or elevational occurrence of Mediterranean-associated taxa, whether Euro-Siberian sites show phenological change without the same compositional response, and whether Irano-Turanian responses depend on the interaction between warming and breeding-site moisture rather than on temperature alone. These predictions require repeated sampling at fixed sites with standardized trap-night units, local weather and habitat measurements, and explicit separation of phenological change from changes in sampling intensity.

Four falsifiable predictions follow from this synthesis. First, if the turnover-dominated between-region pattern persists after method, season, habitat, and effort are equalized, support for genuine biogeographical filtering will increase; if it collapses, source structure is the more parsimonious explanation. Second, if transition provinces are true ecotonal interfaces, standardized surveys should recover greater cross-sector sharing and more intermediate assemblage composition than in core provinces, and repeated surveys should make them sensitive sentinels of climate-associated redistribution; failure of transition status to add explanatory value after effort standardization would argue against that interpretation. Third, if the perfectly nested Mediterranean pattern weakens after balanced replication, its present form is primarily a documentation artifact rather than a stable ecological hierarchy. Fourth, if pathogen-detection and host-feeding evidence hotspots redistribute after balanced, integrated surveillance, their present geography is strongly conditioned by research intensity rather than transmission potential. These hypotheses turn the principal uncertainties—sampling bias, transition-zone structure, climate sensitivity, and sparse vector evidence—into an explicit prospective research design rather than treating unavailable covariates as if they had been measured.

## 5. Conclusions

Reported *Culicoides* richness in Türkiye is strongly conditioned by uneven documentation and is consistent with a substantial Wallacean shortfall. Sampling-effort proxies were the strongest recoverable predictors of provincial richness, whereas an independent regional richness effect was not supported after accounting for sampling effort. Province maps and regional totals should therefore be interpreted primarily as evidence-coverage summaries rather than estimates of true diversity. The different numerical totals reported in this review represent distinct evidence and analytical layers rather than conflicting estimates. Community analyses suggest turnover-dominated regional differentiation, but the literature-derived data cannot separate genuine biogeographical structure from sampling and source effects. Vector-related evidence is likewise too sparse and heterogeneous for species-level risk ranking or predictive transmission mapping.

Future work should prioritize standardized, seasonally replicated sampling in poorly documented provinces and transition zones, using common trap-night units, voucher-linked morphological and molecular identification, and integrated abundance, host-feeding, pathogen, climate, habitat, and livestock data. Such surveillance would allow direct tests of whether apparent regional turnover, Mediterranean nestedness, narrow recorded ranges, and current vector-evidence hotspots persist after sampling effort and methodology are standardized. Until comparable data are available, this synthesis provides a transparent national baseline for identifying surveillance gaps and designing coordinated studies across Türkiye and adjacent biogeographical systems.

## Figures and Tables

**Table 1:** Evidence and analytical data hierarchy used in the synthesis. The layers are linked but not interchangeable; each supports a defined set of analyses and excludes stronger inferences not justified by the source structure. Layer sizes and definitions are detailed in Supplementary File S1 (Sheet S11).

Evidence Layer	Source and Scope	Analytical Unit	Size or Character	Used for	Does Not Support
Eligible evidence-report corpus	Türkiye-specific *Culicoides* reports identified by database or citation searching	Report (with family-level provenance control)	58 reports; 43 operational study/data-source families	Review scope, evidence provenance, narrative synthesis	A single richness estimate or vector-risk analysis
National taxonomic reconciliation	Checklist, new country record, plus one separately identified national monograph	Taxon × source	72 working total; 74 contextual total	National faunistic interpretation	Locality richness, incidence estimation, or abundance
Taxon-record compilation	Core faunistic, ecological, molecular, host-feeding, and pathogen studies	Taxon × geographic record	1164 records; 62 taxa	Distribution and evidence-coverage summaries	Standardized true richness, abundance, or detection probability
Primary province dataset	Primary province-resolved records	Province × taxon incidence	18 provinces; 43 taxa	Effort associations, GLM, PERMANOVA, β-diversity, richness diagnostics	National fauna totals or causal environmental mechanisms
All-explicit province dataset	Primary, secondary, and historical explicit province records	Province × taxon incidence	33 provinces; 45 taxa	Sensitivity analyses	Equivalence with the primary province dataset
Vector-evidence layer	Pathogen screening and blood-meal studies	Study, pool, or taxon evidence	Sparse and heterogeneous	Evidence-gap synthesis and surveillance priorities	Vector competence, relative species risk, or predictive risk mapping
Cross-border contextual layer	National/broad regional inventories; localized studies assessed separately	Country or source	Heterogeneous	Contextual biogeographical discussion	Pooled transboundary richness or formal country ranking

**Table 2:** Reported *Culicoides* species richness in Türkiye based on national checklist literature, new country records, and subsequent taxonomic reassessments.

Reference	Year	Scope	Reported Species Richness	Main Contribution
Dik et al. [9]	2006	Historical national review	57 species	First comprehensive national synthesis; 54 localities and major regional gaps identified
Korkmaz et al. [10]	2021	Updated national checklist and survey	71 species	Updated checklist; ten new country records and expanded national coverage
Turgut [11]	2022	Subsequent new country record	72 species	First Turkish record of *C. clastrieri* from Sinop Province
Dik & Turgut [12] *	2025	National faunistic monograph and taxonomic reassessment	74 species	Recognition of *C. indistinctus* and *C. vitreipennis* as distinct species in Türkiye

* Dik and Turgut [12] was identified separately through targeted searching and citation tracking and was used only for national taxonomic reconciliation. It was not added to the 58-report eligible evidence corpus or to the province-level analytical matrices.

**Table 3:** Summary of vector-related evidence from Türkiye used for epidemiological interpretation. Evidence is classified by theme, region or province, taxa or target group, main result, and interpretation. Field detection of viral nucleic acid in *Culicoides* pools is interpreted as supportive surveillance evidence, not as confirmation of vector competence.

Evidence Theme	Region/Province	Taxa or Target Group	Main Result	Interpretation
AKAV field detection	Eastern Mediterranean: Hatay, Kahramanmaraş, Osmaniye	*C. schultzei* complex; *C. longipennis*; *C. circumscriptus*	AKAV nucleic acid detected in *Culicoides* pools	Field detection only; not confirmed vector competence [7]
SBV/BTV field screening	Tekirdağ, Northwest Türkiye	*C. festivipennis* and other *Culicoides* spp.	SBV RNA detected in one *C. festivipennis* pool; BTV negative	Field detection only; supports targeted surveillance [16]
BEF outbreak-associated survey	Southern/Southeastern Türkiye	11 species; *C. schultzei* dominant; *C. imicola* second dominant	BEFV detected in cattle blood; *Culicoides* pools PCR-negative	Outbreak-associated survey without entomological detection [15]
Host-feeding evidence	24 cattle farms in 20 provinces	Seven species/species complexes	Only cattle blood detected in 69 pools from engorged females	Host-feeding context; farm-biased sampling design [17]
AKAV molecular characterization	Aydın, Aegean Türkiye	*C. imicola*	Complete 702 nt N-gene characterized from a previously AKAV-positive vector specimen collected in 2017	Molecular epidemiological evidence; not a new field-detection or vector-competence result [49]

**Table 4:** Reported *Culicoides* taxon richness across the three principal biogeographical regions of Türkiye, based on explicit province-level records. Values represent reported or observed richness influenced by uneven sampling effort and are not estimates of true regional diversity. The Konya breeding site dataset is excluded as a secondary ecological record.

Biogeographical Region	Contributing Provinces	Reported Taxa	Region-Exclusive Taxa	Notes
Euro-Siberian	18	41	22	Most intensively sampled; Black Sea and Thrace
Mediterranean	7	21	1	Includes Eastern Mediterranean vector-survey provinces
Irano-Turanian	7	12	0	Least sampled; interior steppe plateau (Konya excluded)
All regions (shared core)	—	9	—	Taxa recorded in all three regions

**Table 5:** Evidence-gap framework for vector-related inference from published Turkish *Culicoides* studies. Field nucleic-acid detection supports surveillance association but not vector competence; negative screening applies only to the sampled conditions; and host-feeding evidence is interpreted within the cattle-farm sampling frame. Source studies are cited in the “Available Turkish evidence” column.

Evidence Domain	Available Turkish Evidence	Supported Inference	Unsupported Inference	Critical Data Gap
AKAV field detection	Seven positive pools: *C. schultzei* complex (5), *C. longipennis* (1), and *C. circumscriptus* (1) in the Eastern Mediterranean [7]	Field association and local AKAV circulation	Experimental vector competence or comparative species risk	Individual infection status, salivary-tissue evidence, abundance-adjusted prevalence, and repeated sampling
AKAV molecular characterization	Complete 702 nt N-gene characterization of one previously AKAV-positive *C. imicola* specimen collected in Aydın in 2017 [49]	Molecular epidemiological characterization of Turkish *Culicoides*-associated AKAV material	A new independent field-detection event or evidence of vector competence	Specimen-level linkage to the original diagnostic detection, repeated vector sampling, and tissue-specific infection evidence
SBV/BTV field screening	SBV RNA in one pool containing one unengorged *C. festivipennis* female; BTV negative in the tested Tekirdağ material [16]	An isolated SBV field detection and BTV non-detection under the survey conditions	National vector status, absence of regional BTV risk, or relative transmission potential	Replicated species-resolved screening, explicit pool denominators, abundance, and competence data
BEFV outbreak survey	20,845 *Culicoides* specimens in 155 groups were BEFV-negative; 13/35 cattle blood samples were positive [15]	Outbreak context and non-detection in the sampled *Culicoides* pools	Absence of a vector role or absence of transmission in the region	Repeated abundance-linked screening across outbreak stages and candidate species
Host-feeding evidence	1225 engorged females in 69 pools from 24 cattle farms/20 provinces; cattle blood detected in all pools [17]	Cattle feeding within the cattle-farm sampling frame	General host preference or geographical differences in host use	Multi-host, habitat-balanced, province-resolved sampling with host availability
Integrated risk prediction	No harmonized abundance, trap-night, infection prevalence, climate, livestock-density, or host-availability dataset [7,15,16,17,49]	Identification of missing covariates and surveillance priorities	Predictive risk mapping, regional relative-risk ranking, or abundance-weighted vector potential	Joint vector-host-pathogen-environment sampling with shared spatial and seasonal units

## Data Availability

The compiled literature-based *Culicoides* dataset for Türkiye, including national checklist data, 1164 taxon records extracted at locality, province, or habitat resolution, historical provenance links, vector-related evidence, regional and provincial summaries, map datasets, and manuscript tables, is provided as Supplementary File S1. The PRISMA 2020 flow diagram, PRISMA 2020 checklist, and full list of the 58 eligible reports included in the review are available as Supplementary Files S2–S4; Supplementary File S4 also identifies the 43 operational study/data-source families used to prevent double counting of shared datasets or field programs and documents report-level provenance and eligibility decisions. No new field or experimental data were generated in this study. All source-derived data were traced to the eligible original reports wherever possible; secondary syntheses used for historical verification are identified as such in the provenance layer. The Python 3.11 scripts used for spatial analysis, figure generation, and statistical calculations are available from the corresponding author upon reasonable request. The source-by-source sampling-design audit documenting methodological comparability and missing effort denominators is provided in Supplementary File S1 (Sheet S24).

## Supplementary Materials

The following supporting information can be downloaded at: https://www.mdpi.com/article/10.3390/insects17080858/s1, File S1: Compiled literature-based *Culicoides* dataset for Türkiye, including source roles, national checklist data, 1164 taxon records extracted at locality, province, or habitat resolution, the historical occurrence-to-original-report provenance crosswalk and provenance summary, sampling-method fields, vector-related evidence, regional and provincial summaries, biogeographical classification, transition-province documentation, incidence matrices, effort models, richness diagnostics, sensitivity analyses, the study-level generalized linear mixed model, and β-diversity partitioning; File S2: PRISMA 2020 flow diagram; File S3: PRISMA 2020 reporting checklist; File S4: the 58 eligible reports, their consolidation into 43 operational study/data-source families, and report-level provenance and eligibility documentation; references [7,10,11,13,14,15,16,17,20,23,43,44,45,46,47,49,50,51,52,53,60,75,76,77,78,79,80,81,82,83,84,85,86,87,88,89,90,91,92,93,94,95,96,97,98,99,100,101,102,103,104,105,106,107,108,109,110,111] are cited therein. The additional national monograph used only for taxonomic reconciliation is cited separately in the main reference list. The study-level sampling-design audit of ten principal analytical sources plus one historical verification/source-tracing source is provided in Supplementary File S1 (Sheet S24). Full PERMDISP diagnostics, including bias-adjusted and unadjusted results, permutation settings, and the fixed random seed, are provided in Supplementary File S1 (Sheet S18).

## Abbreviations

The following abbreviations are used in this manuscript:

βJNE	Jaccard nestedness-resultant component
βJTU	Jaccard turnover component
βJAC	Total Jaccard dissimilarity
AKAV	Akabane virus
BEF	Bovine ephemeral fever
BEFV	Bovine ephemeral fever virus
BLV	Bovine leukemia virus
BTV	Bluetongue virus
CI	Confidence interval
GLM	Generalized linear model
ICE	Incidence-based coverage estimator
PCR	Polymerase chain reaction
RNA	Ribonucleic acid
SBV	Schmallenberg virus
PERMANOVA	Permutational Multivariate Analysis of Variance
PERMDISP	Permutational Analysis of Multivariate Dispersions
PCoA	Principal Coordinates Analysis
PPRV	Peste des petits ruminants virus
PRISMA	Preferred Reporting Items for Systematic Reviews and Meta-Analyses

## References

[1] Mellor P.S., Boorman J., Baylis M. (2000). *Culicoides* Biting Midges: Their Role as Arbovirus Vectors. Annu. Rev. Entomol..

[2] Kampen H., Werner D. (2023). Biting Midges (Diptera: Ceratopogonidae) as Vectors of Viruses. Microorganisms.

[3] Cuéllar A.C., Jung Kjær L., Baum A., Stockmarr A., Skovgard H., Nielsen S.A., Andersson M.G., Lindström A., Chirico J., Lühken R. (2018). Monthly Variation in the Probability of Presence of Adult *Culicoides* Populations in Nine European Countries and the Implications for Targeted Surveillance. Parasites Vectors.

[4] Carpenter S., Szmaragd C., Barber J., Labuschagne K., Gubbins S., Mellor P. (2008). An Assessment of *Culicoides* Surveillance Techniques in Northern Europe: Have We Underestimated a Potential Bluetongue Virus Vector?. J. Appl. Ecol..

[5] McDermott E.G., Mayo C.E., Gerry A.C., Laudier D., MacLachlan N.J., Mullens B.A. (2015). Bluetongue Virus Infection Creates Light Averse *Culicoides* Vectors and Serious Errors in Transmission Risk Estimates. Parasites Vectors.

[6] Ducheyne E., De Deken R., Bécu S., Codina B., Nomikou K., Mangana-Vougiaki O., Georgiev G., Purse B.V., Hendickx G. (2007). Quantifying the Wind Dispersal of *Culicoides* Species in Greece and Bulgaria. Geospat. Health.

[7] Dağalp S.B., Dik B., Doğan F., Farzani T.A., Ataseven V.S., Acar G., Şahinkesen İ., Özkul A. (2021). Akabane Virus Infection in Eastern Mediterranean Region in Turkey: *Culicoides* (Diptera: Ceratopogonidae) as a Possible Vector. Trop. Anim. Health Prod..

[8] Sellers R.F., Pedgley D.E. (1985). Possible Windborne Spread to Western Turkey of Bluetongue Virus in 1977 and of Akabane Virus in 1979. J. Hyg..

[9] Dik B., Yağci Ş., Linton Y. (2006). A Review of Species Diversity and Distribution of *Culicoides* Latreille, 1809 (Diptera: Ceratopogonidae) in Turkey. J. Nat. Hist..

[10] Korkmaz C., Alten B., Erol U., Deniz A. (2021). Updated Checklist of *Culicoides* Latreille (Diptera: Ceratopogonidae) of Turkey with Ten New Records. J. Vector Ecol..

[11] Turgut F. (2022). Morphological Characteristics of Two *Culicoides* Latreille Biting Midges (Diptera, Ceratopogonidae) with A New Record for Turkish Fauna. Trak. Univ. J. Nat. Sci..

[12] Dik B., Turgut F. (2025). Türkiye Culicoides (Diptera: Ceratopogonidae) Faunası.

[13] Turgut F., Kiliç A.Y. (2015). The Ceratopogonidae (Insecta: Diptera) Fauna of the Central Black Sea Region in Turkey. Turk. J. Zool..

[14] Turgut F. (2018). Contributions to *Culicoides* Latreille, 1809 (Diptera: Ceratopogonidae) Fauna of Sinop Province. Sak. Univ. J. Sci..

[15] Dik B., Muz D., Muz M.N., Uslu U. (2014). The Geographical Distribution and First Molecular Analysis of *Culicoides* Latreille (Diptera: Ceratopogonidae) Species in the Southern and Southeastern Turkey during the 2012 Outbreak of Bovine Ephemeral Fever. Parasitol. Res..

[16] Muz D., Dik B., Muz M.N. (2023). The Investigation of *Culicoides* (Diptera: Ceratopogonidae) Species and Bluetongue Virus and Schmallenberg Virus in Northwest Türkiye. Trop. Anim. Health Prod..

[17] Deniz A., Korkmaz Ç., Bedir H., Vatansever Z., İtik Ekinci A., Sezer O., Erol U., Tuncer S. (2023). Host Preferences of Vector *Culicoides* (Diptera, Ceratopogonidae, *Culicoides* Latreille) Species in Türkiye. Kafkas Univ. Vet. Fak. Derg..

[18] McGregor B.L., Shults P.T., McDermott E.G. (2022). A Review of the Vector Status of North American *Culicoides* (Diptera: Ceratopogonidae) for Bluetongue Virus, Epizootic Hemorrhagic Disease Virus, and Other Arboviruses of Concern. Curr. Trop. Med. Rep..

[19] Hortal J., De Bello F., Diniz-Filho J.A.F., Lewinsohn T.M., Lobo J.M., Ladle R.J. (2015). Seven Shortfalls That Beset Large-Scale Knowledge of Biodiversity. Annu. Rev. Ecol. Evol. Syst..

[20] Doğan F., Dik B., Bilge-Dağalp S., Farzani T.A., Ataseven V.S., Acar G., Şahinkesen İ., Özkul A. (2022). Prevalance of Schmallenberg Orthobunyavirus (SBV) Infection in Sampled Ruminants in Turkey’s Eastern Mediterranean Region between 2015 and 2017. Res. Vet. Sci..

[21] Bilge Dağalp S., Dik B., Doğan F., Ataseven V.S., Sahinkesen İ., Fedai T., Aslan Ç.U., Özkul A. (2026). Prevalence of Bluetongue Virus (BTV) Infection in Various Domestic Ruminant Species in the Eastern Mediterranean Region, Türkiye. Trop. Anim. Health Prod..

[22] Page M.J., McKenzie J.E., Bossuyt P.M., Boutron I., Hoffmann T.C., Mulrow C.D., Shamseer L., Tetzlaff J.M., Akl E.A., Brennan S.E. (2021). The PRISMA 2020 Statement: An Updated Guideline for Reporting Systematic Reviews. BMJ.

[23] Uslu U., Dik B. (2007). Description of Breeding Sites of *Culicoides* Species (Diptera: Ceratopogonidae) in Turkey. Parasite.

[24] Atalay I. (1986). Vegetation formations of Turkey. Trav. Inst. Geogr. Reims.

[25] Davis P.H., Mill R.R., Tan K. (1988). Flora of Turkey and the East Aegean Islands.

[26] Davis P.H., Cullen J., Coode M.J.E. (1965). Flora of Turkey and the East Aegean Islands.

[27] McCullagh P., Nelder J.A. (2019). Generalized Linear Models.

[28] Ver Hoef J.M., Boveng P.L. (2007). Quasi-Poisson vs. Negative Binomial Regression: How Should We Model Overdispersed Count Data?. Ecology.

[29] Bolker B.M., Brooks M.E., Clark C.J., Geange S.W., Poulsen J.R., Stevens M.H.H., White J.-S.S. (2009). Generalized Linear Mixed Models: A Practical Guide for Ecology and Evolution. Trends Ecol. Evol..

[30] Jaccard P. (1912). The Distribution of the Flora in the Alpine Zone. New Phytol..

[31] Anderson M.J. (2001). A New Method for Non-parametric Multivariate Analysis of Variance. Austral Ecol..

[32] Gower J.C. (1966). Some Distance Properties of Latent Root and Vector Methods Used in Multivariate Analysis. Biometrika.

[33] Anderson M.J. (2006). Distance-Based Tests for Homogeneity of Multivariate Dispersions. Biometrics.

[34] Anderson M.J., Walsh D.C.I. (2013). PERMANOVA, ANOSIM, and the Mantel Test in the Face of Heterogeneous Dispersions: What Null Hypothesis Are You Testing?. Ecol. Monogr..

[35] Baselga A. (2010). Partitioning the Turnover and Nestedness Components of Beta Diversity. Glob. Ecol. Biogeogr..

[36] Baselga A. (2012). The Relationship between Species Replacement, Dissimilarity Derived from Nestedness, and Nestedness. Glob. Ecol. Biogeogr..

[37] Chao A. (1987). Estimating the Population Size for Capture-Recapture Data with Unequal Catchability. Biometrics.

[38] Burnham K.P., Overton W.S. (1978). Estimation of the Size of a Closed Population When Capture Probabilities Vary among Animals. Biometrika.

[39] Smith E.P., Van Belle G. (1984). Nonparametric Estimation of Species Richness. Biometrics.

[40] Lee S.-M., Chao A. (1994). Estimating Population Size Via Sample Coverage for Closed Capture-Recapture Models. Biometrics.

[41] Heltshe J.F., Forrester N.E. (1983). Estimating Species Richness Using the Jackknife Procedure. Biometrics.

[42] Chao A., Jost L. (2012). Coverage-based Rarefaction and Extrapolation: Standardizing Samples by Completeness Rather than Size. Ecology.

[43] Dik B., Kuçlu Ö., Öztürk R. (2017). *Culicoides* Latreille, 1809 (Diptera: Ceratopogonidae) Species in the Western Black Sea Region of Turkey, New Records for the Turkish Fauna. Turk. J. Vet. Anim. Sci..

[44] Turgut F. (2018). Samsun İli *Culicoides* Latreille, 1809 (Diptera: Ceratopogonidae) Faunasına Katkılar. Gaziosmanpaşa Bilimsel Araşt. Derg..

[45] Dik B., Yaman M., Uslu U. (2010). *Culicoides* species (Latreille, 1809) (Diptera: Ceratopogonidae) in Hatay Province. Kafkas Univ. Vet. Fak. Derg..

[46] Deniz A., Öncel T., Patakakis M.J. (2010). Species Composition of *Culicoides* Latreille, 1809 (Diptera: Ceratopogonidae) in Thrace Region of Turkey. Kafkas Univ. Vet. Fak. Derg..

[47] Yildirim A., Dik B., Duzlu O., Onder Z., Ciloglu A., Yetismis G., Inci A. (2019). Genetic Diversity of *Culicoides* Species within the *Pulicaris* Complex (Diptera: Ceratopogonidae) in Turkey Inferred from Mitochondrial COI Gene Sequences. Acta Trop..

[48] Petersen F.T., Meier R. (2003). Testing Species-Richness Estimation Methods on Single-Sample Collection Data Using the Danish Diptera. Biodivers. Conserv..

[49] Pekmez K., Kaplan M., Çağırgan A.A., Arslan F. (2026). The Complete Nucleoprotein Gene Molecular Characterization of Akabane Virus in Türkiye. Arch. Virol..

[50] Doğan F., Bilge Dağalp S., Dik B., Farzani T.A., Alkan F. (2020). Detection of Genotype 1 Bovine Leukemia Virus from a *C. schultzei* Pool: Do *Culicoides* spp. Have a Role on the Transmission of Bovine Leukemia Virus?. Infect. Genet. Evol..

[51] Yeşilöz H., Önder Z., Yıldırım A. (2025). Molecular Characterization of *Culicoides* Species and Their Vector Potentials for Haemosporidia Infections in the İzmir Region of Türkiye. Turk. Parazitol. Derg..

[52] Şevik M., Oz M.E. (2019). Detection of Peste Des Petits Ruminants Virus RNA in *Culicoides imicola* (Diptera: Ceratopogonidae) in Turkey. Vet. Ital..

[53] Ozan E., Albayrak H., Gumusova S., Bolukbas C.S., Kurt M., Pekmezci G.Z., Beyhan Y.E., Kadi H., Kaya S., Aydin I. (2019). A Study on the Identification of Five Arboviruses from Hematophagous Mosquitoes and Midges Captured in Some Parts of Northern Turkey. J. Arthropod Borne Dis..

[54] Borkent A., Dominiak P. (2020). Catalog of the Biting Midges of the World (Diptera: Ceratopogonidae). Zootaxa.

[55] Brugger K., Rubel F. (2013). Characterizing the Species Composition of European *Culicoides* Vectors by Means of the Köppen-Geiger Climate Classification. Parasites Vectors.

[56] Sprygin A.V., Fiodorova O.A., Babin Y.Y., Elatkin N.P., Mathieu B., England M.E., Kononov A.V. (2014). *Culicoides* Biting Midges (Diptera, Ceratopogonidae) in Various Climatic Zones of Russia and Adjacent Lands. J. Vector Ecol..

[57] Pudar D., Petrić D., Allène X., Alten B., Ayhan N., Cvetkovikj A., Garros C., Goletić T., Gunay F., Hlavackova K. (2018). An Update of the *Culicoides* (Diptera: Ceratopogonidae) Checklist for the Balkans. Parasites Vectors.

[58] Abdigoudarzi M. (2016). Some New Records of *Culicoides* Species (Diptera: Ceratopogonidae) from Iran. J. Arthropod Borne Dis..

[59] Patakakis M.J., Papazahariadou M., Wilson A., Mellor P.S., Frydas S., Papadopoulos O. (2009). Distribution of *Culicoides* in Greece. J. Vector Ecol..

[60] Nolan D.V., Dallas J.F., Piertney S.B., Mordue A.J. (2008). Incursion and Range Expansion in the Bluetongue Vector *Culicoides imicola* in the Mediterranean Basin: A Phylogeographic Analysis. Med. Vet. Entomol..

[61] Balenghien T., Alexander N., Arnþórsdóttir A.L., Bisia M., Blackwell A., Bødker R., Bourquia M., Boutsini S., Carpenter S., Colenutt C. (2020). *Culicoides* Abundance Distribution Models for Europe and Surrounding Regions. Open Health Data.

[62] Fetene E., Teka G., Dejene H., Mandefro D., Teshome T., Temesgen D., Negussie H., Mulatu T., Jaleta M.B., Leta S. (2022). Modeling the Spatial Distribution of *Culicoides* Species (Diptera: Ceratopogonidae) as Vectors of Animal Diseases in Ethiopia. Sci. Rep..

[63] Jacquot M., Nomikou K., Palmarini M., Mertens P., Biek R. (2017). Bluetongue Virus Spread in Europe Is a Consequence of Climatic, Landscape and Vertebrate Host Factors as Revealed by Phylogeographic Inference. Proc. R. Soc. B..

[64] Bibard A., Martinetti D., Giraud A., Picado A., Chalvet-Monfray K., Porphyre T. (2025). Quantitative Risk Assessment for the Introduction of Bluetongue Virus into Mainland Europe by Long-Distance Wind Dispersal of *Culicoides* spp.: A Case Study from Sardinia. Risk Anal..

[65] Moshavernia S., Alipour H. (2026). Bluetongue Virus in Middle Eastern Ruminants: Vector Evidence, Surveillance Gaps, and Research Priorities. Trop. Anim. Health Prod..

[66] Arzumanyan M., Tarzyan N., Rulik B., Pont A. (2025). A Preliminary Checklist of the Diptera of Armenia. ZooKeys.

[67] Belkharchouche M., Berchi S., Mathieu B., Rakotoarivony I., Duhayon M., Baldet T., Balenghien T. (2020). Update of the *Culicoides* (Diptera: Ceratopogonidae) Species Checklist from Algeria with 10 New Records. Parasites Vectors.

[68] Bourquia M., Garros C., Rakotoarivony I., Gardès L., Huber K., Boukhari I., Delécolle J.-C., Baldet T., Mignotte A., Lhor Y. (2019). Update of the Species Checklist of *Culicoides* Latreille, 1809 Biting Midges (Diptera: Ceratopogonidae) of Morocco. Parasites Vectors.

[69] Filatov S., Szadziewski R. (2017). Annotated Checklist and Distribution of *Culicoides* Biting Midges of Ukraine (Diptera: Ceratopogonidae). J. Nat. Hist..

[70] Braverman Y., Boorman J., Kremer M. (1976). Faunistic List of *Culicoides* (Diptera, Ceratopogonidae) from Israel. Cah. ORSTOM Sér. Entomol. Méd. Parasitol..

[71] Navai S. (1974). Studies of the *Culicoides* of Iran. Ann. Parasitol. Hum. Comp..

[72] Wieczorek J., Bloom D., Guralnick R., Blum S., Döring M., Giovanni R., Robertson T., Vieglais D. (2012). Darwin Core: An Evolving Community-Developed Biodiversity Data Standard. PLoS ONE.

[73] Sanders C.J., Shortall C.R., England M., Harrington R., Purse B., Burgin L., Carpenter S., Gubbins S. (2019). Long-Term Shifts in the Seasonal Abundance of Adult *Culicoides* Biting Midges and Their Impact on Potential Arbovirus Outbreaks. J. Appl. Ecol..

[74] Bowen J.M., Tilston N.L. (2026). Climate Change and the Growing Threat of Bunyaviruses. J. Virol..

[75] Kieffer J.J. (1918). Chironomides d’Afrique et d’Asie conservés au Museum National Hongrois de Budapest. Ann. Hist.-Nat. Mus. Natl. Hung..

[76] Edwards F.W., Oldroyd H., Smart J. (1939). British Blood-Sucking Flies.

[77] Leclercq M. (1966). Contribution à l’étude des diptères suceurs de sang en Turquie (Culicidae, Ceratopogonidae, Muscidae, Hippoboscidae). Bull. Rech. Agron. Gembloux.

[78] Navai S. (1977). Biting Midges of the Genus *Culicoides* (Diptera: Ceratopogonidae) from Southwest Asia. Ph.D. Thesis.

[79] Jennings M. (1982). *Culicoides* Collected in Turkey in October 1981. Mosq. News.

[80] Jennings M., Boorman J.P.T., Ergün H. (1983). *Culicoides* from Western Turkey in Relation to Bluetongue Disease of Sheep and Cattle. Rev. Élev. Méd. Vét. Pays Trop..

[81] Dik B. (1989). Konya ve Çevresinde Bulunan *Culicoides* (Diptera: Ceratopogonidae) Türleri Üzerine Araştırmalar. Ph.D. Thesis.

[82] Burgu İ., Urman H.K., Akça Y., Yonguç A., Mellor P.S., Hamblin C., Walton T.E., Osburn B.I. (1992). Serologic Survey and Vector Surveillance for Bluetongue in Southern Turkey. Bluetongue, African Horse Sickness, and Related Orbiviruses: Proceedings of the Second International Symposium.

[83] Dik B., Dinçer Ş. (1992). Studies on *Culicoides* (Diptera: Ceratopogonidae) Species around Konya (Turkey). Doğa-Tr. J. Vet. Anim. Sci..

[84] Dik B. (1993). Adana, İçel ve Antalya Yörelerinde Bulunan *Culicoides* Latreille, 1809 (Diptera: Ceratopogonidae) Türlerinin Tespiti. Türk Vet. Hekim. Derg..

[85] Yılmaz H. (1994). Elazığ Yöresinde Bulunan *Culicoides* (Diptera: Ceratopogonidae) Türleri Üzerine Araştırmalar. Ph.D. Thesis.

[86] Eren H., Yağcı Ş., Dinçer Ş. (1995). Ankara’da Bulunan *Culicoides* (Diptera: Ceratopogonidae) Türleri. Ank. Üniv. Vet. Fak. Derg..

[87] Yılmaz H., Dumanlı N. (1995). Elazığ ve Yöresinde İlk Kez Saptanan Bazı *Culicoides* (Diptera: Ceratopogonidae) Türleri. Fırat Üniv. Sağlık Bil. Derg. (Vet.).

[88] Dik B. (1996). Ege Bölgesi *Culicoides* (Diptera: Ceratopogonidae) Türlerinin Tespiti. Acta Parasitol. Turc..

[89] Yılmaz H., Dumanlı N. (1997). Elazığ Yöresinde *Culicoides* (Diptera: Ceratopogonidae) Türleri Üzerinde Araştırmalar. Acta Parasitol. Turc..

[90] Yağcı Ş., Eren H., Dinçer Ş. (1999). Aydın (Umurlu)’da Saptanan Bazı Nematocera (Diptera) Türleri. Acta Parasitol. Turc..

[91] Eren H., İnci A. (2002). Bursa (Gemlik)’da Saptanan *Culicoides* (Diptera: Ceratopogonidae) Türleri. Acta Parasitol. Turc..

[92] Uslu U. (2003). Konya Yöresinde *Culicoides* (Diptera: Ceratopogonidae) Türlerinin Üreme Yerleri Üzerine Araştırmalar. Ph.D. Thesis.

[93] Tilki N., Dik B. (2003). Farklı Renkteki Işıkların *Culicoides* (Diptera: Ceratopogonidae) Türlerinin Yakalanmaları Üzerine Etkileri. Acta Parasitol. Turc..

[94] Uslu U., Dik B. (2004). Seasonal Distribution of Species *Culicoides* (Diptera: Ceratopogonidae) in Konya Province. Eurasian J. Vet. Sci..

[95] Dik B., Karatepe M., Karatepe B., Yağcı Ş. (2006). Niğde Yöresi *Culicoides* Latr., 1809 (Diptera: Ceratopogonidae) Türleri. Türkiye Parazitol. Derg..

[96] Dik B., Ergül R. (2006). Nocturnal Flight Activities of *Culicoides* (Diptera: Ceratopogonidae) Species in Konya. Türkiye Parazitol. Derg..

[97] Uslu U., Dik B. (2006). Vertical Distribution of *Culicoides* Larvae and Pupae. Med. Vet. Entomol..

[98] Dik B., Kurt M., Aydın İ. (2008). Karadeniz Bölgesindeki *Culicoides* (Diptera: Ceratopogonidae) Türleri Üzerinde Bir Araştırma. Bornova Vet. Kontrol Araşt. Enst. Derg..

[99] Uslu U., Dik B. (2010). Chemical Characteristics of Breeding Sites of *Culicoides* Species (Diptera: Ceratopogonidae). Vet. Parasitol..

[100] Turgut F. (2011). Orta Karadeniz Bölgesi Ceratopogonidae (Insecta: Diptera) Faunasının Araştırılması. Ph.D. Thesis.

[101] Uslu U., Dik B. (2011). Seasonal Distribution of Immature Stages of *Culicoides* (Diptera: Ceratopogonidae) Species. Eurasian J. Vet. Sci..

[102] Dik B., Yavru S., Uslu U., Yapıcı O., Esin E.I. (2012). Determination of *Culicoides* Species (Diptera: Ceratopogonidae) as Suspect Vectors of Epizootic Haemorrhagic Disease and Bluetongue Viruses in Southern and Western Anatolia. Rev. Méd. Vét..

[103] Şevik M., Doğan M. (2017). Epidemiological and Molecular Studies on Lumpy Skin Disease Outbreaks in Turkey during 2014–2015. Transbound. Emerg. Dis..

[104] Şevik M. (2017). Molecular and Serological Survey of Akabane Virus Infection in Sheep in the Mediterranean Region of Turkey. Small Rumin. Res..

[105] Yeşilöz H. (2017). İzmir Yöresinde Culicoides Türlerinin Moleküler Karakterizasyonu ve Haemosporidia Enfeksiyonları Yönünden Vektörlük Potansiyellerinin Araştırılması. Ph.D. Thesis.

[106] Yavru S., Dik B., Bulut O., Uslu U., Yapıcı O., Kale M., Avcı O. (2018). New *Culicoides* Vector Species for BTV Transmission in Central and Central West of Anatolia. Annu. Res. Rev. Biol..

[107] Mignotte A., Garros C., Gardès L., Balenghien T., Duhayon M., Rakotoarivony I., Tabourin L., Poujol L., Mathieu B., Ibañez-Justicia A. (2020). The Tree That Hides the Forest: Cryptic Diversity and Phylogenetic Relationships in the Palaearctic Vector *Obsoletus/Scoticus* Complex (Diptera: Ceratopogonidae) at the European Level. Parasites Vectors.

[108] Gökçe M. (2021). Batı Karadeniz Bölgesinde *Culicoides* (Diptera: Ceratopogonidae) Cinsi Sineklerin Yoğunluk Haritasının Oluşturulması ve Mekânsal Analizi. Master’s Thesis.

[109] Turgut F., Küçük Ş. (2022). *Culicoides* Biting Midges (Diptera: Ceratopogonidae) of Sarıkum Nature Reserve Area (Sinop-Turkey). Munis Entomol. Zool..

[110] Korkmaz Ç. (2022). Türkiye’de *Culicoides* (Diptera: Ceratopogonidae) Türlerinin Dağılımı ve Ekolojik Yaklaşımlar. Ph.D. Thesis.

[111] Buğdaycı İ., Gökçe M., Nacar F. (2024). Spatial Analysis of *Culicoides* Biting Midges (Diptera: Ceratopogonidae) in the Western Black Sea Region. Uzaktan Konumsal Araştırma ve Analiz.

