# Supplementary material for: Reported Distribution, Species Richness, and Sampling Bias of Culicoides (Diptera: Ceratopogonidae) in Türkiye: A Literature-Based Synthesis with a Biogeographical Framework
Source: Insects. 2026 Aug 17;17(8):858. doi: 10.3390/insects17080858 (PMC13513888; doi:10.3390/insects17080858)
Supplement: Supplementary file 1 [file insects-17-00858-s001.zip › Supplementary_File_S3_PRISMA_2020_Checklist.pdf]

PRISMA 2020 Reporting Checklist

PRISMA-guided review: Reported Distribution, Species Richness and Sampling Bias of *Culicoides* (Diptera: Ceratopogonidae) in Türkiye: A Literature-Based Synthesis with a Biogeographical Framework

Reporting checklist for the manuscript and Supplementary Files S1–S4 (August 2026).

From: Page MJ, et al. The PRISMA 2020 statement: an updated guideline for reporting systematic reviews. BMJ 2021;372:n71. doi: 10.1136/bmj.n71

Section	Item	Checklist item	Location in manuscript
**Title**	1	Identify the report as a review using a transparent PRISMA-guided reporting framework.	Title page
**Abstract**	2	See the PRISMA 2020 for Abstracts checklist.	Abstract
**Introduction**	3	Rationale: Describe the rationale for the review in the context of existing knowledge.	Introduction
	4	Objectives: Provide an explicit statement of the objective(s) or question(s) the review addresses.	Introduction
**Methods**	5	Eligibility criteria: Specify the inclusion and exclusion criteria for the review and how studies were grouped for the syntheses.	Section 2.2. Eligibility was restricted to reports generating new Türkiye-specific *Culicoides* evidence from Turkish specimens/field observations or through an original analysis of Türkiye-specific *Culicoides* data. Publication type was not an exclusion criterion. Theses and other grey-literature reports were eligible when they met the same original-evidence criterion. Narrative reviews and secondary compilations were excluded as evidence reports. Multiple eligible reports describing the same underlying field programme or dataset were retained for provenance but consolidated into a single study/data-source family.
	6	Information sources: Specify all databases, registers, websites, organisations, reference lists and other sources searched or consulted to identify studies. Specify the date when each source was last searched or consulted.	Section 2.2; Supplementary File S1 (Sheet S12_Search_Strategy); Supplementary File S2. Web of Science (n = 94), Scopus (n = 57), and Google Scholar (n = 219) were searched with no date restriction; searches were completed through August 2026. Reference/citation searching of historical synthesis sources was also used to identify original reports.
	7	Search strategy: Present the full search strategies for all databases, registers and websites, including any filters and limits used.	Section 2.2; Supplementary File S1 (Sheet S12_Search_Strategy); Supplementary File S2. The search expression was "*Culicoides*" AND ("Turkey" OR "Turkiye") with no date restriction. Supplementary File S4 documents the 58 eligible reports, their study/data-source family assignments, and report-level provenance/eligibility information.
	8	Selection process: Specify the methods used to decide whether a study met the inclusion criteria of the review, including how many reviewers screened each record and each report retrieved, whether they worked independently, and if applicable, details of automation tools used in the process.	Section 2.2. Initial title-and-abstract screening and full-text/report eligibility assessment were performed by the first author against predefined inclusion and exclusion criteria. The second author subsequently and independently verified the study-selection decisions at both stages, including the final inclusion/exclusion decisions and the eligible evidence corpus. Any disagreements were resolved by discussion until consensus. No automation tool made screening or eligibility decisions.
	9	Data collection process: Specify the methods used to collect data from reports, including how many reviewers collected data from each report, whether they worked independently, any processes for obtaining or confirming data from study investigators, and if applicable, details of automation tools used in the process.	Section 2.2; Section 2.6; Author Contributions. Source data were curated into Supplementary File S1 by the first author and checked against the cited reports. The second author subsequently and independently verified the final manuscript, supplementary files, study-selection decisions, and overall consistency. Historical occurrence records recovered through Dik et al. (2006) were reassigned to the cited original report(s) in the provenance layer; the review itself was retained only as a verification/source-tracing source. No automation tool performed autonomous source-data extraction.
	10a	Data items: List and define all outcomes for which data were sought. Specify whether all results that were compatible with each outcome domain in each study were sought (e.g. for all measures, time points, analyses), and if not, the methods used to decide which results to collect.	Section 2.3; Section 2.4.1
	10b	List and define all other variables for which data were sought (e.g. participant and intervention characteristics, funding sources). Describe any assumptions made about any missing or unclear information.	Section 2.3; Section 2.4.1
	11	Study risk of bias assessment: Specify the methods used to assess risk of bias in the included studies, including details of the tool(s) used, how	Section 2.3; Section 4.6; Supplementary File S1 (Sheet S24_Study_Design_Audit). No common validated risk-of-bias instrument was applicable across the heterogeneous faunistic, ecological,

		many reviewers assessed each study and whether they worked independently, and if applicable, details of automation tools used in the process.	surveillance, molecular and host-feeding designs. Instead, a structured source-level study-design audit documented sampling period, collection method, spatial coverage, material/sample totals, denominator availability, identification approach, analytical role and major comparability limitations. The audit covers ten principal analytical sources plus one historical national review retained only for verification/citation tracing. No numerical risk-of-bias score was assigned.
	12	Effect measures: Specify for each outcome the effect measure(s) (e.g. risk ratio, mean difference) used in the synthesis or presentation of results.	Effect measures included Spearman rank correlation coefficients (ρ), generalized linear model coefficients and model fit summaries, PERMANOVA F statistics, R^2 values and permutation p values, PERMDISP F statistics and permutation p values, PCoA axis eigenvalue percentages, and incidence-based richness estimates (Chao2, first- and second-order jackknife, and ICE) reported with 95% confidence intervals, estimated sample coverage, and the turnover and nestedness-resultant components of Jaccard dissimilarity.
	13a	Synthesis methods: Describe the processes used to decide which studies were eligible for each synthesis (e.g. tabulating the study intervention characteristics and comparing against the planned groups for each synthesis).	Section 2.2; Section 2.4
	13b	Describe any methods required to prepare the data for presentation or synthesis, such as handling of missing summary statistics, or data conversions.	Section 2.3
	13c	Describe any methods used to tabulate or visually display results of individual studies and syntheses.	Section 2.4.1; Section 3.4
	13d	Describe any methods used to synthesise results and provide a rationale for the choice(s). If meta-analysis was performed, describe the model(s), method(s) to identify the presence and extent of statistical heterogeneity, and software package(s) used.	Sections 2.4–2.6. Reported richness was analysed using Spearman correlations and Poisson generalized linear models; study-level non-independence was examined with a Poisson generalized linear mixed model. Assemblage structure was described using Jaccard dissimilarity, PERMANOVA, PCoA, and turnover/nestedness partitioning. Homogeneity of multivariate dispersion was assessed with permutational analysis of multivariate dispersions (PERMDISP) using distances to group spatial medians and permutation tests. Chao2, first- and second-order jackknife, ICE, and sample coverage were used as exploratory richness diagnostics. Python scripts were executed and verified by the authors and are available from the corresponding author upon reasonable request.
	13e	Describe any methods used to explore possible causes of heterogeneity among study results (e.g. subgroup analysis, meta-regression).	Sections 2.4, 3.5 and 3.6. Heterogeneity in reported richness and assemblage composition was explored using sampling-effort variables, dominant biogeographical regions and transition-province flags.
	13f	Describe any sensitivity analyses conducted to assess robustness of the synthesised results.	Section 2.4 and Supplementary File S1. Five analytical scenarios, including the baseline model, were reported: the primary 18-province dataset; all 33 provinces with explicit records; the 33-province dataset excluding Konya, the most intensively sampled province; the subset excluding provinces classified as transitional; and the primary dataset excluding transition provinces. Across all estimable GLM scenarios, the sampling-effort coefficient remained positive with a confidence interval excluding zero, and the biogeographical-region term was never significant after adjustment for effort.
	14	Reporting bias assessment: Describe any methods used to assess risk of bias due to missing results in a synthesis (arising from reporting biases).	Section 2.4.3; Section 4.6
	15	Certainty assessment: Describe any methods used to assess certainty (or confidence) in the body of evidence for an outcome.	No formal certainty assessment was conducted. Confidence in the findings was discussed qualitatively in relation to sampling heterogeneity, geographical coverage, and limitations of the literature-based evidence.
**Results**	16a	Study selection: Describe the results of the search and selection process, including findings from automation tools if used, in the text and with a flow diagram (PRISMA 2020 flow diagram).	Section 2.2; Supplementary File S1 (Sheet S12_Search_Strategy); Supplementary File S2. A total of 370 records/candidates were identified (Web of Science = 94; Scopus = 57; Google Scholar = 219). After removal of 139 duplicate or already represented records, 231 records were screened and 175 were excluded at title/abstract. Fifty-six reports were retrieved and assessed at full-text/report level; six were excluded, leaving 50 database-derived eligible reports. Reference/citation searching identified eight additional original reports; all eight were assessed and eligible. The final evidence corpus therefore contains 58 eligible reports, consolidated into 43 operational study/data-source families.
	16b	Cite studies that might appear to meet the inclusion criteria, but which were excluded, and explain why they were excluded.	Section 2.2; Supplementary File S4 (sheet Full_Text_Exclusions_6), with Supplementary Files S1 and S2. Six reports were excluded after full-text/report assessment: five secondary

			review/synthesis/background reports and one report without demonstrated new Türkiye-specific *Culicoides* evidence. Supplementary File S4 identifies all six excluded reports and provides their exclusion categories and reasons. Title/abstract screening excluded 175 records under the predefined eligibility criteria. Secondary syntheses used only for verification or citation tracing (e.g., Dik et al. 2006) were not counted as eligible evidence reports.
	17	Study characteristics: Cite each included study and present its characteristics.	Section 3; Supplementary Files S1 and S4. Supplementary File S4 lists all 58 eligible reports with discovery route, evidence path, analytical role, provenance, and consolidation into 43 operational study/data-source families; it also provides the harmonized final search-selection summary.
	18	Risk of bias in studies: Present assessments of risk of bias for each included study.	Section 3; Section 4 / Limitations; Supplementary File S1 (Sheet S24_Study_Design_Audit). Formal numerical risk-of-bias scoring was not applied. Source-level design limitations and comparability constraints were documented systematically and interpreted alongside sampling-effort, spatial-coverage and evidence-layer limitations.
	19	Results of individual studies: For all outcomes, present, for each study: (a) summary statistics for each group (where appropriate) and (b) an effect estimate and its precision (e.g. confidence/credible interval), ideally using structured tables or plots.	Section 3 and Supplementary File S1. Individual and grouped study contributions are summarized through source tables, locality records, province summaries, evidence categories and statistical/ordination outputs.
	20a	Results of syntheses: For each synthesis, briefly summarise the characteristics and risk of bias among contributing studies.	Sections 3.5, 3.6 and 3.10
	20b	Present results of all statistical syntheses conducted. If meta-analysis was done, present for each the summary estimate and its precision and measures of statistical heterogeneity.	Sections 3.5–3.6 and Supplementary File S1. Reported outputs include sampling-effort correlations, Poisson GLM and GLMM results, PERMANOVA, PERMDISP, PCoA, Jaccard turnover/nestedness partitioning, Chao2, jackknife estimators, ICE, sample coverage, and associated confidence intervals or permutation statistics where applicable.
	20c	Present results of all investigations of possible causes of heterogeneity among study results.	Sections 3.5 and 3.6. Variation among provinces and biogeographical regions was evaluated using sampling-effort variables, dominant-region grouping and assemblage-composition analyses.
	20d	Present results of all sensitivity analyses conducted to assess the robustness of the synthesised results.	Supplementary File S1. Five analytical scenarios, including the baseline model, were evaluated: the primary 18-province dataset; all 33 provinces with explicit records; the 33-province dataset excluding Konya; the subset of all explicit provinces excluding transition provinces; and the primary dataset excluding transition provinces. Across the estimable scenarios, the sampling-effort effect remained positive and the biogeographical-region term remained non-significant after adjustment for effort.
	21	Reporting biases: Present assessments of risk of bias due to missing results (arising from reporting biases) for each synthesis assessed.	Discussion. Reporting and publication biases are considered through the Wallacean shortfall framework, uneven sampling effort, incomplete province-level coverage and the distribution of evidence types.
	22	Certainty of evidence: Present assessments of certainty (or confidence) in the body of evidence for each outcome assessed.	Discussion / Limitations. No formal certainty assessment was conducted. Confidence in the findings was discussed qualitatively in relation to sampling heterogeneity, geographical coverage, and limitations of the literature-based evidence.
**Discussion**	23a	Discussion: Provide a general interpretation of the results in the context of other evidence.	Section 4 (Sections 4.1–4.5)
	23b	Discuss any limitations of the evidence included in the review.	Section 4 / Limitations
	23c	Discuss any limitations of the review processes used.	Section 4 / Limitations
	23d	Discuss implications of the results for practice, policy, and future research.	Section 4 / Conclusions

Other	24	Registration and protocol: Provide registration information for the review, including register name and registration number, or state that the review was not registered.	This review was not pre-registered. As a PRISMA-guided review of observational ecological and faunistic literature rather than a clinical intervention review, prospective registration in PROSPERO was not applicable and therefore not pursued.
	25	Support: Describe sources of financial or other support for the review, and the role of the funders or sponsors in the review.	Funding section. This research received no external funding.
	26	Competing interests: Declare any competing interests of review authors.	Conflict of interest
	27	Availability of data, code and other materials: Report which of the following are publicly available and where they can be found: template data collection forms; data extracted from included studies; data used for all analyses; analytic code; any other materials used in the review.	Supplementary Files S1–S4. S1 provides the evidence and analytical layers, including 1,164 taxon-level records, provenance documentation, study-design audit, incidence matrices, models and sensitivity analyses; S2 provides the PRISMA 2020 flow diagram; S3 is this checklist; and S4 provides the 58 eligible reports, 43 study/data-source families, corpus reconciliation, and the harmonized final search-selection summary. Analysis and figure-generation scripts are available from the corresponding author upon reasonable request.
